# Characterization and validation of *Entamoeba histolytica* pantothenate kinase as a novel anti-amebic drug target

**DOI:** 10.1016/j.ijpddr.2018.02.004

**Published:** 2018-03-01

**Authors:** Arif Nurkanto, Ghulam Jeelani, Takehiro Yamamoto, Yoshiko Naito, Takako Hishiki, Mihoko Mori, Makoto Suematsu, Kazuro Shiomi, Tetsuo Hashimoto, Tomoyoshi Nozaki

**Affiliations:** a Graduate School of Life and Environmental Sciences, University of Tsukuba, Tsukuba, Japan; b Department of Parasitology, National Institute of Infectious Diseases (NIID), Tokyo, Japan; c Department of Biomedical Chemistry, Graduate School of Medicine, The University of Tokyo, Japan; d Research Center for Biology, Indonesia Institute of Sciences (LIPI), Cibinong, Indonesia; e Department of Biochemistry, School of Medicine, Keio University, Tokyo, Japan; f Kitasato Institute for Life Sciences, Kitasato University, Tokyo, Japan; g Clinical and Translational Research Center, Keio University School of Medicine, Japan

**Keywords:** *Entamoeba histolytica*, Amebiasis, Coenzyme A, Pantothenate kinase, Gene silencing, Drug development

## Abstract

The Coenzyme A (CoA), as a cofactor involved in >100 metabolic reactions, is essential to the basic biochemistry of life. Here, we investigated the CoA biosynthetic pathway of *Entamoeba histolytica* (*E. histolytica*), an enteric protozoan parasite responsible for human amebiasis. We identified four key enzymes involved in the CoA pathway: pantothenate kinase (PanK, EC 2.7.1.33), bifunctional phosphopantothenate-cysteine ligase/decarboxylase (PPCS-PPCDC), phosphopantetheine adenylyltransferase (PPAT) and dephospho-CoA kinase (DPCK). Cytosolic enzyme PanK, was selected for further biochemical, genetic, and phylogenetic characterization. Since *E. histolytica* PanK (EhPanK) is physiologically important and sufficiently divergent from its human orthologs, this enzyme represents an attractive target for the development of novel anti-amebic chemotherapies. Epigenetic gene silencing of *PanK* resulted in a significant reduction of PanK activity, intracellular CoA concentrations, and growth retardation *in vitro*, reinforcing the importance of this gene in *E. histolytica*. Furthermore, we screened the Kitasato Natural Products Library for inhibitors of recombinant EhPanK, and identified 14 such compounds. One compound demonstrated moderate inhibition of PanK activity and cell growth at a low concentration, as well as differential toxicity towards *E. histolytica* and human cells.

## Highlights

• Pantothenate kinase (PanK) is essential in *Entamoeba histolytica* parasite.
• *PanK* gene silencing caused a significant growth defect, CoA level, and PanK activity.
• PanK represents an attractive target for new drug development.
• Potential inhibitors have been found against *E. histolytica* PanK.

## Graphical abstract

**Figure:**
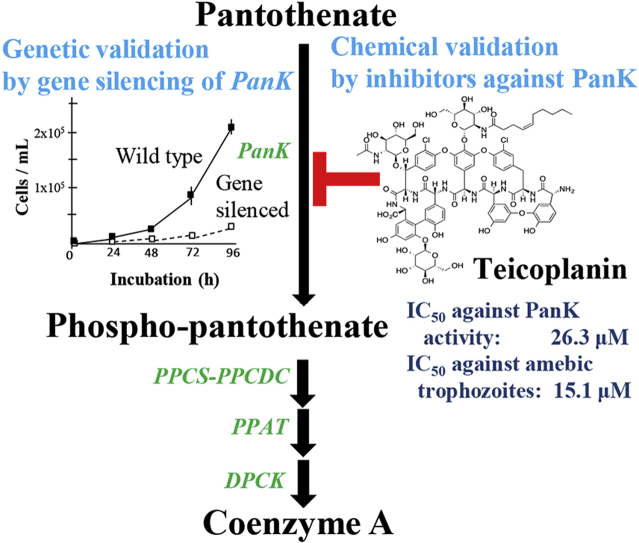

## 1 Introduction

Coenzyme A (CoA) is an essential cofactor in all living organisms as an acyl group carrier and carbonyl-activating group involved in more than 100 cellular reactions (Begley et al., 2001); it is estimated to be a cofactor used in 9% of identified enzymatic reactions (Strauss, 2010). CoA biosynthesis is considered to be an essential and universal pathway of the majority of prokaryotes and eukaryotes (Leonardi et al., 2005b). In general, CoA participates in fatty acid metabolism, the tricarboxylic acid cycle and numerous other intermediary metabolic reactions (Abiko, 1975). CoA is synthesized from pantothenate (vitamin B_5_), cysteine, and ATP (Jackowski, 1996; Leonardi et al., 2005b). Most of the eukaryotes are unable to synthesize pantothenic acid and thus rely on an external supply.

*Entamoeba histolytica* is the protozoan agent responsible for human amebiasis, an infectious disease causing dysentery and amebic liver abscesses, responsible for 100,000 deaths annually throughout the world. It represents the third most common parasitic cause of death, after malaria and schistosomiasis (Stanley, 2003; Ali and Nozaki, 2007; Ralston and Petri, 2011). The elaborate pathogenesis of this parasite is well documented (Espinosa-Cantellano and Martínez-Palomo, 2000; Nozaki and Bhattacharya, 2015). Metronidazole has, for decades, been the most effective drug in the treatment of amebiasis despite its known side effects and low efficacy against asymptomatic cyst carriers. Moreover, resistance, virulence and host immune response to metronidazole treatment in amebiasis have been reported in some countries (Griffin, 1973; Pittman and Pittmann, 1974; Johnson, 1993; Koch et al., 1997; Ali and Nozaki, 2007). The molecular target of metronidazole has been well described as a key metabolic enzyme, pyruvate: ferredoxin oxidoreductase, which is involved in acetyl CoA production from pyruvate as part of central energy metabolism. Identification and characterization of novel drug targets unique to *E. histolytica* are therefore needed to design better therapeutics against amebiasis.

Here we investigate the CoA biosynthetic pathway of *E. histoltyica.* We identified four enzymes, including one bifunctional enzyme, involved in this pathway. We further characterized one of these enzymes, pantothenate kinase (PanK, EC 2.7.1.33), with biochemical and reverse genetic approaches. Moreover, we identified *E. histolytica* PanK inhibitors by screening the Kitasato Natural Products Library against EhPanK recombinant enzyme. Taken together, we demonstrate that the CoA biosynthetic pathway, in general, and PanK, specifically, represents a rational and novel drug target against amebiasis.

## 2 Materials and methods

### 2.1 Organisms, cultivation, and chemicals

Trophozoites of *E. histolytica* clonal strains HM-1: IMSS cl 6 and G3 (Bracha et al., 2006) were maintained axenically in Diamond's BI-S-33 medium at 35.5 °C as described previously (Diamond et al., 1978). Trophozoites were continuously maintained in mid-log phase after inoculation of one-thirtieth to one-twelfth of the total culture volume. *Escherichia coli* BL21 (DE3) strain was obtained from Invitrogen (Carlsbad, CA, USA). Magnesium-free ATP was procured from DiscoverX (Fremont, CA, USA). Ni^2+^-NTA agarose was procured from Novagen (Darmstadt, Germany). Lipofectamine and geneticin (G418) were procured from Invitrogen. Chemicals to evaluate metals in PanK activity assay were procured from Wako (Tokyo, Japan). All other chemicals of analytical grade were procured from Sigma-Aldrich (Tokyo, Japan) unless otherwise stated.

### 2.2 Production of PanK gene-silenced strain

In order to construct a plasmid for epigenetic gene silencing of *E. histolytica* (Bracha et al., 2006; Zhang et al., 2011) *PanK* (*EhPanK*), a fragment corresponding to a 430 bp 5′ open reading frame of *EhPanK* gene was amplified by PCR from cDNA using the following primer set (sense primer, 5′-CAG**AGGCCT**ATGTCTCAACCATCCCATTCT-3′ and antisense primer, 5′-AAT**GAGCTC**TCTGAAGATTACCAATCCCATAAA-3′). These oligonucleotides contained StuI and SacI restriction sites (shown in bold). The amplified product was digested with StuI and SacI, and ligated into the StuI and SacI double digested psAP2-Gunma construct (Husain et al., 2011) to synthesize a *EhPanK* gene silencing plasmid. The G3 strain trophozoites were transformed with an empty vector as a control and the silencing plasmid was transfected by liposome-mediated transfection as previously described (Nozaki et al., 1999). Transformants were initially selected in the presence of 1 μg/mL geneticin gradually increased to 10 μg/mL.

### 2.3 Reverse transcriptase PCR

RNA was extracted from approximately 1 × 10^6^ trophozoites of *EhPanK* gene silenced (PanK gs) and control transformant strains using TRIzol reagent (Ambion, Life Technologies) as previously described (Chomczynski and Mackey, 1995). DNase treatment was performed using DNase I (Invitrogen) to exclude genomic DNA. RNA quantity was determined by measuring the absorbance at 260 nm with a NanoDrop ND-1000 UV–Vis spectrophotometer (NanoDrop Technologies, Wilmington, DE, USA). Approximately one μg total RNA was used for cDNA synthesis using First-Stand cDNA Synthesis (Superscript^®^ III, Invitrogen) with reverse transcriptase and oligo (dT) primers according to manufacturer's instructions. The cDNA product was diluted 10-fold and PCR reactions were carried out in 50 μl, using the primer pair (Sense primer, 5′-ATGTCTCAACCATCCCATTC-3′ and antisense primer, 5′-TTACATTAGTTCTTCTTCATACTC-3′). The PCR conditions were: 98 °C, 10 s; 55 °C, 1 min; and 72 °C, 1 min; 20–25 cycles. The PCR products obtained were resolved by agarose gel electrophoresis.

### 2.4 Quantitative real-time (qRT) PCR

Relative levels of steady-state mRNA of the following genes were measured using qRT-PCR: *PanK* (EHI_183060), bifunctional phosphopantothenate-cysteine ligase/decarboxylase (*PPCS-PPCDC*, EHI_164490), phosphopantetheine adenylyltransferase (*PPAT*, EHI_006680), dephospho CoA kinase 1 and 2 (*DPCK1,* EHI_040840; and *DPCK2*, EHI_155780), and RNA polymerase II gene (EHI_056690) as a control. Each 20 μL reaction contained 10 μL 2X Fast SYBR Green Master Mix (Applied Biosystems, Foster City, CA, USA), 0.6 μL each of 10 μM sense and antisense primers, 5 μL 10x diluted cDNA, and nuclease-free water. PCR was performed using the StepOne Plus Real-Time PCR System (Applied Biosystems, Foster City, CA, USA) with the following cycling conditions: enzyme activation at 95 °C for 20 s, followed by 40 cycles of denaturation at 95 °C for 3 s and annealing-extension at 60 °C for 30 s. All reactions were carried out in triplicate, including cDNA-minus controls. The amount of the steady-state mRNA of each target gene was determined by the ΔCt method with RNA polymerase II as a reference gene (Livak and Schmittgen, 2001). The mRNA expression level of each gene in the transformant was expressed relative to that in the control transfected with psAP2.

### 2.5 Production of whole lysates from *E. histolytica* trophozoites

Approximately 1 × 10^6^ trophozoites were harvested 48 h after initiation of culture and washed with 2% glucose in 1X phosphate buffer saline (PBS) three times. Cells were counted and resuspended in 500 μL homogenization buffer (50 mM Tris-HCl, pH 7.5, 250 mM sucrose, 50 mM NaCl) supplemented with 1 mM phenylmethylsulfonyl fluoride (PMSF) and 0.5 mg/mL E-64 (Peptide Institute, Osaka, Japan). Cells were disrupted mechanically by a Dounce homogenizer and kept on ice for 30 min with intermittent vortexing followed by centrifugation at 500 × g for 30 min at 4 °C for removing the insoluble cellular debris. The supernatant, representing total cell lysate, was carefully collected. Protein concentrations were spectrophotometrically determined by the Bradford method using bovine serum albumin as a standard as previously described (Bradford, 1976).

### 2.6 Enzyme assays and quantitation of CoA in cell lysates

PanK and DPCK activities in the cell lysate were measured with coupled assays using ADP Hunter™ Plus Assay kit (DiscoverX, US) according to the manufacturer's instructions. Briefly, fluorescence intensities were continuously measured to estimate the formation of resorufin at 37 °C by excitation at 530 nm and emission at 590 nm in a 25 μL reaction mixture [10 mM MgCl_2_, 15 mM HEPES, 20 mM NaCl, 1 mM EGTA, 0.02% tween 20, 0.1 mg/mL β-globulin, 2 mM pantothenate or 2 mM dephospho CoA for PanK or DPCK, respectively, 0.1 mM ATP, 2 μL of cell lysate (∼5 μg protein)]. Kinetic data were estimated by curve fitting with the Michaelis–Menten equation using GraphPad Prism (GraphPad Software Inc., San Diego, USA). This experiment was repeated three times in triplicate with proteins isolated from two independent extractions, and kinetic values are presented as the means ± S.E. for three independent kinetic assays.

Concentrations of CoA in the cell lysate were measured using the CoA assay kit (BioVision, CA, USA) according to manufacturer's instructions. CoA at 0.05–1 nmole was used to produce a standard curve to determine the amounts of CoA in lysates. Experiment was conducted in triplicate, and repeated three times on three different days.

### 2.7 Monitoring of growth kinetics

Trophozoite cultures were continuously maintained in mid-log phase as described previously, and placed on ice for 5 min to detach cells from the glass surface. Cells were collected by centrifugation at 500 × g for 5 min at room temperature. After discarding the spent medium, the pellet was re-suspended in 1 mL of BI-S-33 medium. Cell densities were estimated on a haemocytometer. Approximately 10,000 trophozoites were inoculated in 6 mL fresh BI-S-33 medium. Cultures were examined every 24 h for 5 days.

### 2.8 Genome-wide survey of enzymes involved in CoA biosynthesis in the *E. histolytica* genome

Putative genes encoding PanK, PPCS-PPCDC, PPAT, and DPCK were identified in the genome of HM-1:IMSS in the *E. histolytica* genome database (AmoebaDB, http://amoebadb.org/amoeba/) using the blastp online search tool (protein-protein BLAST). Human or bacterial orthologs (Human PanK1α/NP_683878.1, *E. coli* bifunctional CoaBC/WP_089667520.1, Human bifunctional COASY/AAL50813.1) retrieved from non-redundant protein sequences (nr) database of National Center for Biotechnology Information (NCBI, http://www.ncbi.nlm.nih.gov/) were used as query sequences. Pathway analysis was conducted using Kyoto Encyclopedia of Genes and Genomes database (KEGG, https://www.genome.jp/kegg). The steady-state mRNA level of each gene in the trophozoite stage was examined using our previous array data as an independent experiment in both HM1: IMSS cl6 (Penuliar et al., 2012, 2015) and G3 strains (Nakada-Tsukui et al., 2012; Furukawa et al., 2012, 2013). Then using *Entamoeba invadens* orthologs, we compared the mRNA expression levels of these genes during various timepoints during the encystation stage (De Cádiz et al., 2013).

### 2.9 Phylogenetic analysis of PanK

Putative orthologs of PanK from a variety of organisms were retrieved by a blastp search of non-redundant protein sequences (nr) database of National Center for Biotechnology Information (NCBI, http://www.ncbi.nlm.nih.gov/) using the EhPanK sequence (XP_001913460) as a query. To comprehensively retrieve all orthologs from representative organisms in the variety of major taxa, we carried out a blastp search against each taxonomic group as shown in Table S1. In each blastp analysis, a list of selected sequences was made with an E-value less than 1 × 10^−10^ in pairwise alignments with EhPanK. Based on the lists for all taxonomic groups in Table S1, we selected 81 sequences as a final data set. The Muscle program (Edgar, 2004) in SeaView software package version 4.6.1 (Gouy et al., 2010) was used for sequence alignment. Then 145 unambiguously aligned positions were selected by manual inspection and used for phylogenetic analyses.

The phylogenic data matrices were subjected to analysis with the IQTREE program (Nguyen et al., 2015) to select appropriate models for amino acid sequence evolution. The LG + Γ4 model was found to be the best for analysis. Maximum likelihood (ML) analysis implemented in the RAxML program version 7.2.6 (Stamatakis, 2006) was used to infer the ML tree by applying LG + Γ4 model. In the bootstrap analysis, heuristic tree search was performed with a rapid bootstrap algorithm option (-f) for 100 bootstrap replicates. Bootstrap proportion (BP) values greater than 50 were indicated on the corresponding internal branches of the ML tree drawn with FigTree program Version 1.4.2 (http://tree.bio.ed.ac.uk/software/figtree/).

### 2.10 Production of *E. histolytica* transformant line to express Myc-tagged PanK

The *EhPanK* gene was amplified from cDNA using Ex Taq DNA polymerase (Takara) using the following primer set (sense primer, 5′- GC**CCCGGG**ATGTCTCAACCATCCCATTC -3′ and antisense, 5′- GC**CTCGAG** TTACATTAGTTCTTCTTCATACTC-3′), restriction sites are indicated in bold. An amplified fragment was ligated into SmaI and XhoI double digested pEhEx-Myc expression vector (Nakada-Tsukui et al., 2012) to produce pEhExMyc-PanK. The plasmid was introduced into trophozoites of *E. histolytica* HM-1:IMSS cl6 (Diamond et al., 1978) by liposome-mediated transfection (Nozaki et al., 1999). Transformants were selected and maintained as described previously.

### 2.11 Cell fractionation and immunoblot analysis

Trophozoites of the amoeba transformant expressing Myc-EhPanK and the mock transformant, transfected with pEhEx-Myc, were washed three times with PBS containing 2% glucose. After resuspension in homogenization buffer (50 mM Tris-HCl, pH 7.5, 250 mM sucrose, 50 mM NaCl and 0.5 mg/mL E-64 protease inhibitor), cells were disrupted mechanically by a Dounce homogenizer on the ice, centrifuged at 500 × g for 5 min, and the supernatant was collected to remove unbroken cells. The supernatant fraction was centrifuged at 5000 × g for 10 min to isolate pellet and supernatant fractions. The 5000 × g supernatant fraction was further centrifuged at 100,000 × g for 60 min to produce a 100,000 × g supernatant and pellet fractions. The pellets at each step were further washed twice with homogenization buffer and re-centrifuged at 100,000 × g for 10 min to minimize carryover. Immunoblot analysis was performed using the fractions and anti-Myc mouse monoclonal antibody. Anti-CPBF1 (cysteine protease binding family protein 1) and anti-CS1 (cysteine synthase 1) rabbit antisera were used as organelle membrane and cytosolic markers, respectively.

### 2.12 Production of *EhPanK* recombinant protein

The plasmid was constructed as previously described (Sambrook and Russell, 2001). DNA fragment was amplified from *E. histolytica* cDNA. Primers used were sense, 5′- GCCG**GGATCC**ATGTCTCAACCATCCCATTC -3′ and antisense, 5′- GCCG **GTCGAC**TTACATTAGTTCTTCTTCATACTC-3’. Bold letters indicate BamHI and SalI restriction sites. PCR was performed with primeSTAR HS DNA polymerase (Takara) and the following parameters: initial incubation at 98 °C for 30 s; followed by the 30 cycles of denaturation at 98 °C for 10 s; annealing at 55 °C for 30 s; and elongation at 72 °C for 1 min; and a final extension at 72 °C for 7 min. The PCR fragment was digested with BamHI and SalI, purified with Wizard^®^ SV Gel and PCR Clean-up System (Promega). The fragment was cloned into BamHI and SalI double digested pCOLD1™ histidine-tag vector (Takara) to finally produce pCOLD1-EhPanK. The nucleotide sequence of the engineered plasmid was verified by sequencing.

pCOLD1-EhPanK was introduced into *E. coli* BL21(DE3) cells via heat shock at 42 °C for 1 min. *E. coli* BL21 (DE3) harboring pCOLD1-EhPanK was grown at 37 °C in 100 mL of Luria Bertani medium (Invitrogen) in the presence of 100 μg/mL ampicillin (Nacalai Tesque). The overnight culture was used to inoculate 500 mL of fresh medium, and the culture was further continued at 37 °C with shaking at 180 rpm. When A_600_ absorbance reached 0.8, then 0.5 mM isopropyl β-D-thiogalactopyranoside (IPTG) was added, and cultivation was continued for another 24 h at 15 °C. *E. coli* cells from the induced culture were harvested by centrifugation at 5000 × g for 20 min at 4 °C. The cell pellet was washed with PBS, pH 7.4, re-suspended in 20 mL of the lysis buffer (50 mM Tris HCl, pH 8.0, 300 mM NaCl, and 10 mM imidazole) containing 0.1% Triton x 100 (v/v), 100 μg/mL lysozyme, and 1 mM PMSF, and incubated at room temperature for 30 min. Then the mixture was sonicated on ice and centrifuged at 25,000 × g for 15 min at 4 °C. The supernatant was mixed with 1.2 mL of 50% Ni^2+^-NTA His-bind slurry, incubated for 1 h at 4 °C with mild shaking. The recombinant enzyme-bound resin in a column was washed three times with buffer A (50 mM Tris-HCl, pH 8.0, 300 mM NaCl, and 0.1% Triton X-100, v/v) containing 10–50 mM of imidazole. Bound protein was eluted with buffer A containing 100–300 mM imidazole. After the integrity and the purity of recombinant protein were confirmed with 12% SDS-PAGE analysis, followed by Coomassie Brilliant Blue staining, the sample was dialyzed against a 300-fold volume of 50 mM Tris-HCl, 150 mM NaCl, pH 8.0 containing 10% glycerol (v/v) and the Complete Mini protease inhibitor cocktail (Roche, Mannheim, Germany) for 18 h at 4 °C. Pure enzyme was stored at −80 °C with 20% glycerol in small aliquots until use.

### 2.13 Screening of natural compounds for EhPanK inhibitors

We screened 244 compounds from the Kitasato Natural Products Library for inhibition of *Eh*PanK activity. Initially, each compound was dissolved in 50% DMSO and water at a final concentration of 1 mg/mL. Enzymatic reactions were carried out on a black 384-well microtiter plate with a 20 μL reaction mixture composed of 19 μl enzyme mix (100 μM pantothenate, 100 μM ATP, 50 ng of EhPanK recombinant enzyme in kinase buffer (see above) and 1 μl of the individual compounds (final concentration 50 μg/mL or 25–200 μM) at 37 °C for 2 h. After the kinase reaction, ADP was measured using ADP Hunter™ Plus kinase assay kit as described previously. Inhibition values were measured in triplicate.

Compounds that showed more than 50% inhibition at <400 μM were re-tested to confirm that they did not inhibit the enzyme in the coupled assay (pyruvate kinase and pyruvate oxidase) in ADP Hunter™ Plus kinase assay kit. Inhibition of the enzymes used in the coupled assay was examined in the reaction mixture (10 mM MgCl_2_, 15 mM HEPES, 20 mM NaCl, 1 mM EGTA, 0.02% tween 20, 0.1 mg/mL β-globulin, 60 μM pantothenate, 50 μM ATP, 10–50 μM ADP, and 25–200 μM of selected active compounds). No significant change was observed in a range of ADP and inhibitor concentrations, suggesting no inhibition of coupled enzymes by the EhPanK inhibitors. Compounds that did not inhibit enzymes used in the coupled assay were selected as hits for further evaluation, including dose-response, determination of concentration needed for 50% inhibition (IC_50_) of PanK activity as well as both *E. histolytica* and human cell growth.

### 2.14 Measurement of anti-amebic activity of EhPanK inhibitor compounds

Approximately 1 × 10^4^ trophozoites of *E. histolytica* clonal strain HM-1:IMSS cl6 in 200 μl of BI-S-33 medium were dispensed into each well of a 96-well plate and incubated at 35.5 °C for 2 h. Medium was then removed and replaced with 200 μl of BI-S-33 medium that contained various concentrations of each compound. The final concentrations of test compounds are 50–600 μM for cephaloridine, hygromycin A, erythromycin A, and fluorescamine; 5–200 μM for kasugamycin, gardimycin, rosamycin, streptomycin, tirandamycin A, neomycin, and teicoplanin; 0.1–10 μM for *O*-methylnanaomycin A, echinomycin, and trichostatin. The plate was incubated under anaerobic conditions at 35.5 °C for 48 h. After the medium was removed, 100 μl of pre-warmed Opti-MEM I (Life Technologies, Grand Islands, NY, USA) containing one-tenth volume of WST-1 (Roche, Mannheim, Germany) was added. The viability of trophozoites was estimated by measuring absorbance at 450 nm by SpectraMax^®^ Paradigm^®^ (Molecular Devices, CA, USA). Metronidazole was used as positive control at final concentrations of 0.1–10 μM and 0.5% DMSO as negative control. Each assay was performed in triplicate. IC_50_ values were calculated by least squares curve fitting of the dose inhibition curves using GraphPad Prism (GraphPad Software Inc., San Diego, USA).

### 2.15 Evaluation of cytotoxicity against MRC-5 cells

Human fibroblast cells, MRC-5, were cultured on 96-well flat bottom plates at a density of 1.5 × 10^4^ cells/well with 100 μl of MEM medium (Life Technologie) containing 10% fetal bovine serum (Hana-nesco Bio, Tokyo, Japan) and 1% penicillin-streptomycin (Life Technologies). Cells were incubated at 37 °C with 5% CO_2_ for 48 h. Approximately 100 μl of MEM medium containing 5 μl of each compound dissolved in 50% DMSO in water were added to each well. After the plates were incubated for 48 h, approximately 10 μl of WST-8 (Dojindo, Kumamoto, Japan) was added to each well and the plate was incubated at 37 °C with 5% CO_2_ for 2 h. The absorbance was measured at 450 nm by spectrophotometer (SH-9000Lab, Corona Electric, Ibaraki, Japan). Growth of MRC-5 cells was measured in duplicate. Staurosporine (Kitasato Natural Products Library) was used as a positive (cytotoxic) control in a concentration range of 0.5 pM to 0.1 nM IC_50_ values were calculated as described previously.

## 3 Results

### 3.1 Identification of four enzymes involved in CoA biosynthesis in *E. histolytica*

**Fig. 1:**
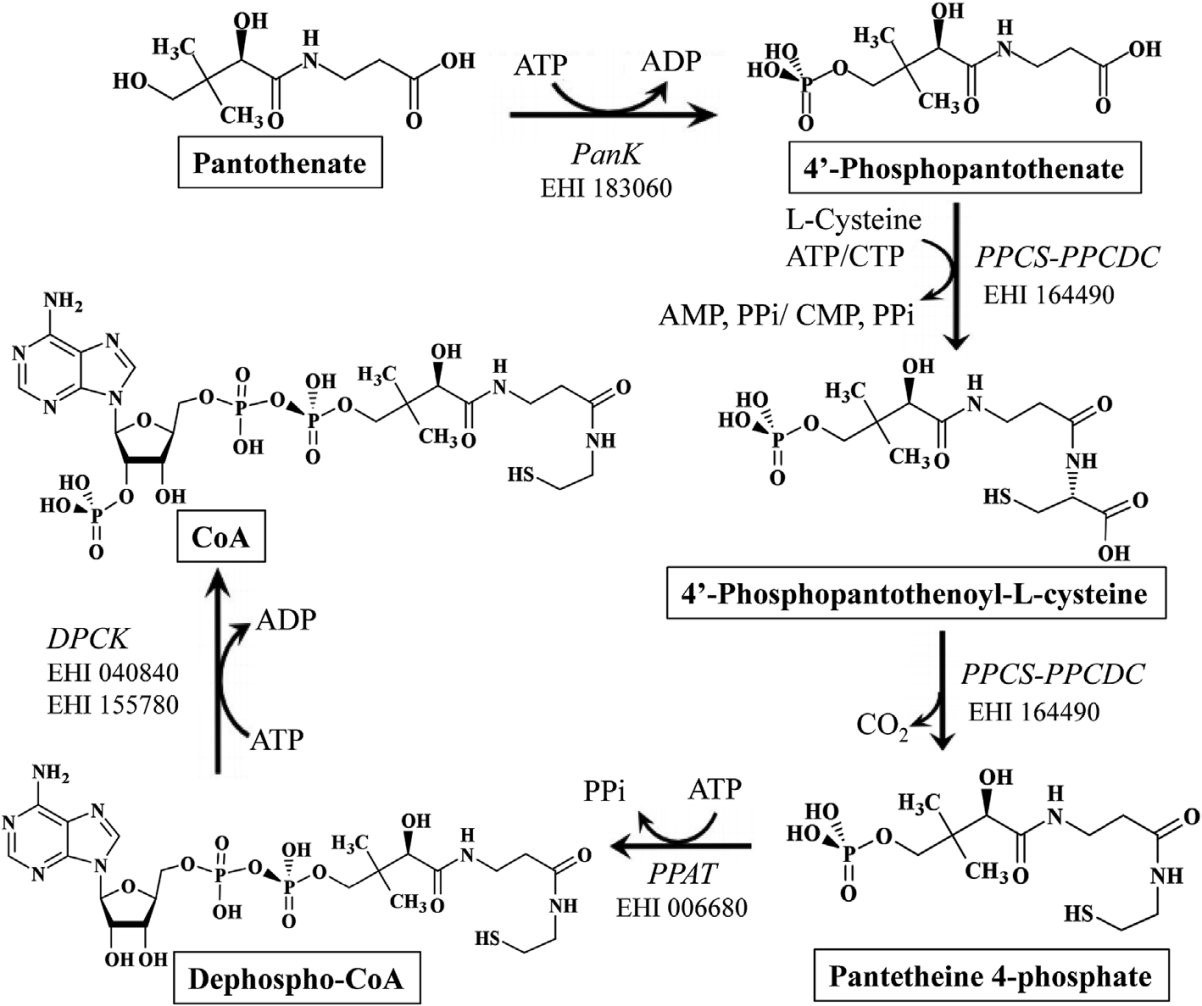
Coenzyme A biosynthetic pathway in *E. histolytica*. ID numbers of individual enzymes in AmoebaDB are also shown. PanK, pantothenate kinase; PPCS-PPCDC, bifunctional phosphopantothenoylcysteine synthetase and phosphopantothenoylcysteine decarboxylase; PPAT, phosphopantetheine adenylyltransferase; and DPCK, dephosphocoenzyme A kinase.

We identified all putative genes involved in CoA biosynthesis in the reference genome of *E. histolytica*: HM-1:IMSS (Fig. 1). Four genes encoding sequential pathway enzymes were identified: pantothenate kinase (EC 2.7.1.33, PanK), bifunctional phosphopantothenate-cysteine ligase/decarboxylase (EC 6.3.2.5/EC 4.1.1.36), phosphopantetheine adenylyltransferase (EC 2.7.7.3), and dephospho CoA kinase (EC 2.7.1.24). We identified a single putative *PanK* gene in each strain (e.g., EHI_183060 in HM-1:IMSS and EHI7A_012650 in HM-1:IMSS-A) in Amoeba Genomics Resources database, AmoebaDB (http://amoebadb.org). The nucleotide sequences of the genes in different strains are 100% identical. Therefore, *E. histolytica* seems to have only one *PanK* gene.

Our previous transcriptome data verified that all four genes are expressed at low to moderate levels (Fig. S1A). The level of mRNA expression relative to RNA polymerase for PanK, PPCS-PPCDC, PPAT, DPCK1, and DPCK2 in trophozoites of the *E. histolytica* clonal strain HM1: IMSS cl6 (Penuliar et al., 2015, 2012) and G3 (Nakada-Tsukui et al., 2012; Furukawa et al., 2012, 2013) strain were 1.2–1.8, 0.5–0.6, 0.3–0.4, 0.04–0.05 and 0.3–0.4, respectively. In this research, we describe biochemical and genetic characterization of PanK; investigation of other enzymes involved in CoA biosynthesis will be described elsewhere. Our previous transcriptomic analysis of *E. invadens* (De Cádiz et al., 2013), as a model for encystation (Donaldson et al., 1975; Kojimoto et al., 2001; Stanley, 2003; Chia et al., 2009), showed that genes encoding these four enzymes were expressed during encystation, but the expression profiles of individual enzymes largely differed (Fig. S1B). The expression profiles of PPCS-PPCDC and DPCK1 were quite peculiar with expression peaking at certain time points (2 h and 24 h, respectively); this subject was, however, not investigated as part of this study.

### 3.2 Features of *EhPanK* and its encoded protein

The 1219-bp *E. histolytica PanK* gene contains two small introns, and is predicted to encode a 403 a.a. polypeptide with a molecular mass of 45.2 kDa and a p*I* value of 5.2. EhPanK showed 99, 96, 83, and 70% sequence identity to PanK from *E. nuttalli*, *E. dispar*, *E. moshkovskii,* and *E. invadens*, respectively. In general, PanK is classified into three groups, type I through III, as determined by primary sequence analysis and kinetic properties (Song and Jackowski, 1993; Rock et al., 2002; Yang et al., 2006). *E. histolytica* PanK is classified as PanK type II, and shows a low level (∼35%) of positional identity to the *A. thaliana* and human orthologs, both of which also belong to type II (Fig. S2 and S3). When compared to human PanK, *Entamoeba* PanK shows the highest similarity to human PanK4 (35%), whereas similarity to other human PanK isotypes (PanK1α, PanK1β, PanK2, and PanK3) is ∼32%. All amino acid residues were inferred to be involved in pantothenate and ATP catalysis and substrate binding include the typical ATP-binding motif have been reported in human PanK1 and PanK3 (Bum et al., 2007). Some conserved glycine residues in the ATP binding motif (Gly^19^ and Gly^321^ in human PanK3; Gly^59^ and Gly^351^ in *E. histolytica*) are indispensable for activity. Mutations of this amino acid have been demonstrated to abolish enzymatic activity because ATP is no longer able to properly bind (Bum et al., 2007). Furthermore, it is understood that phosphorylation is proceeded by an ordered sequential mechanism, with ATP binding preceding pantothenate binding (Leonardi et al., 2005a).

### 3.3 Phylogenetic analysis of EhPanK

We determined the phylogenetic relationship between 81 putative PanK orthologs collected from bacteria and eukaryotes (Table S1, Fig. S2). The majority of eukaryotes possess the *PanK* gene. Mammals (Opisthokonta) and land plants (Viridiplantae) apparently possess twice or more two genes. For instance, four *PanK* genes were identified from *Homo sapiens*, PanK1α and PanK1β (Rock et al., 2002), PanK2 (annotated as a mitochondrial precursor protein) (Hörtnagel et al., 2003; Kotzbauer et al., 2005), and PanK3 (Zhang et al., 2005). Archaea and most bacterial groups do not possess *PanK* homologs that show similarity to *Eh*PanK with the E-value less than 10^−10^. *PanK* homologs were identified, however in Firmicutes, specifically *Bacilli*. During sequence alignment, only the ‘fumble’ domain (PFAM:PFO03630) could be aligned among all 81 sequences, although insertions and deletions were needed for proper alignment due to a high degree of divergence. PanKs from Euglenozoa have an ∼1000-a.a. long N-terminal extension, which contains an exonuclease-endonuclease-phosphatase (EEP) domain in the 300 a.a.-long N-terminal region and a 550 a.a.-long adenylate forming (AF) domain. Some of PanK from *Viridiplantae* have a 300 a.a.-long C-terminal extension, annotated as DUF89 (Domain of Unknown Function 89) (Huang et al., 2016). PanK from other organisms including the genus *Entamoeba* basically contain only a ‘fumble’ domain; neither the EEP/AF nor DUF89 domain is present in PanK from other organisms except that some possess an N-terminal extension that appears to be an organelle targeting sequence.

Based on 145 aligned positions from the ‘fumble’ domain, an optimal ML tree was created with bootstrap proportion (BP) support values (Fig. S2). Eukaryotes and Firmicutes were separated clearly with a 100% BP support value. The Eukaryota clade had monophylies of several taxonomic groups reconstructed, but BP values were generally low except for those of Viridiplantae monophyly (88%) and Haptophyceae monophyly (97%). Four *Entamoeb*a species are monophyletic with 100% BP support, and the *Entamoeba* clade shares a sister group position to the clade of Euglenozoa. However, since BP support values for deep branching patterns were very low, monophyly of Amoebozoa including the *Entamoeba* clade cannot be ruled out. Although this analysis could not precisely infer the phylogenetic position of the *Entamoeba* PanK, it is apparently of a eukaryotic origin.

### 3.4 Expression and purification of recombinant PanK

A soluble EhPanK recombinant protein with a 2.6 kDa histidine tag at the amino terminus was successfully produced using the pCOLD I *E. coli* expression system and purified to homogeneity (>95% as evaluated with Coomassie Brilliant Blue stained SDS-PAGE gel) (Fig. S4A, Table S2). Immunoblot analysis of the purified recombinant protein using His-Tag antibody confirmed the absence of truncation (Fig. S4B). The molecular mass of the purified protein under reducing conditions was consistent with the predicted molecular mass 45.2 kDa excluding the histidine tag. The specific activity of the purified enzyme was estimated to be 1.5 μmol/min/mg when assayed under the standard conditions described in the “Materials and methods” section. EhPanK is robust and catalytically active in broad pH range with maximum activity obtained at pH 6 and 37 °C (Fig. S5A).

### 3.5 Kinetic properties and phosphoryl donor specificities of EhPanK, and effects of metal ions on EhPanK

Table 1 summarizes the apparent *K*_m_, *V*_max_, and *K*_cat_ values for EhPanK using pantothenate and ATP as substrates. EhPanK exhibited hyperbolic saturation kinetics when assayed over the substrate range of 4–256 μM for pantothenate in the presence of 25–100 μM ATP (Fig. S5B) and 1–100 μM ATP in the presence of 8–128 μM pantothenate (Fig. S5C). The apparent *K*_m_ value for pantothenate at saturating ATP concentrations was 53.2 ± 7.1 μM, and the *K*_m_ value for ATP at saturating pantothenate concentrations was 41.4 ± 3.9 μM.

**Table 1:** Kinetic parameters of *E. histolytica* pantothenate kinase.

Substrate	*K*_m_ (μM)	*V*_max_ (μmole/min/mg)	*K*_cat_ (min^−1^)	*K*_cat_/*K*_m_ (min^−1^μM^−1^)
Pantothenate	53.2 ± 7.1	2.79 ± 0.1	126.8 ± 4.5	2.38 ± 0.08
ATP	41.4 ± 3.9	2.84 ± 0.2	129.1 ± 9.2	3.11 ± 0.22

Mean ± SEM of three replicates are shown.

EhPanK utilized various nucleoside triphosphates, ATP, CTP, GTP, UTP, dATP, and polyphosphates, as well as deoxynucleotides as phosphoryl donors, with ATP being the best phosphate donor (Table 2). EhPanK showed an absolute requirement for a free bivalent metal cofactor, with Mg^2+^ as the preferred cation (Table 3).

**Table 2:** Phosphoryl donor specificity of *E. histolytica* pantothenate kinase.

Phosphoryl donor^a^	Relative activity (%)	Phosphoryl donor^a^	Relative activity (%)
ATP	100.0 ± 4.1	CTP	1.4 ± 0.2
GTP	78.8 ± 4.9	Metaphosphate	ND
dATP	19.3 ± 4.3	Hexametaphosphate	ND
Tripolyphosphate	16.9 ± 0.8	Trimetaphosphate	ND
UTP	16.6 ± 1.2	Glucose-6-phosphate	ND
Polyphosphate	16.2 ± 2.5	Phosphoenolpyruvate	ND
TTP	5.4 ± 1.9	None	ND

Assays were performed as described in Materials and methods, in the presence 15 mM HEPES, 20 mM NaCl, 10 mM MgCl_2_, 1 mM EGTA, 0.02% Tween-20, 0.1 mg/mL β−globulins and 0.2 mM pantothenate. Reactions were conducted at 37 °C at pH 6.

The activity is shown in percentage (%) relative to that toward ATP.

ND, not detected.

Mean ± SEM of three replicates are shown.

a The final concentration used was 100 μM.

**Table 3:** Effect of metal ions on the activity of *E. histolytica* pantothenate kinase.

Metal^a^	Relative activity (%)
MgCl_2_	100.0 ± 0.0
CuCl_2_	84.3 ± 4.7
MnCl_2_	83.2 ± 2.0
CoCl_2_	74.2 ± 6.1
FeCl_2_	72.8 ± 1.7
LiCl_2_	34.5 ± 1.8
CaCl_2_	17.0 ± 3.0
ZnCl_2_	3.9 ± 0.7
NiCl_2_	ND
NaCl	ND
KCl	ND
None	ND

Assays were performed as described in Materials and methods, in the presence of 100 mM ATP, 15 mM HEPES, 20 mM NaCl, 1 mM EGTA, 0.02% Tween-20, 0.1 mg/mL β−globulins and 0.2 mM pantothenate. Reactions were conducted at 37 °C at pH 6.0.

The activity is shown in percentage (%) relative to that toward MgCl_2_. ND, not detected.

Mean ± SEM of three replicates are shown.

a The cation final concentration used was 5 mM.

### 3.6 Regulation of EhPanK by CoA, acetyl CoA, and malonyl CoA

**Fig. 2:**
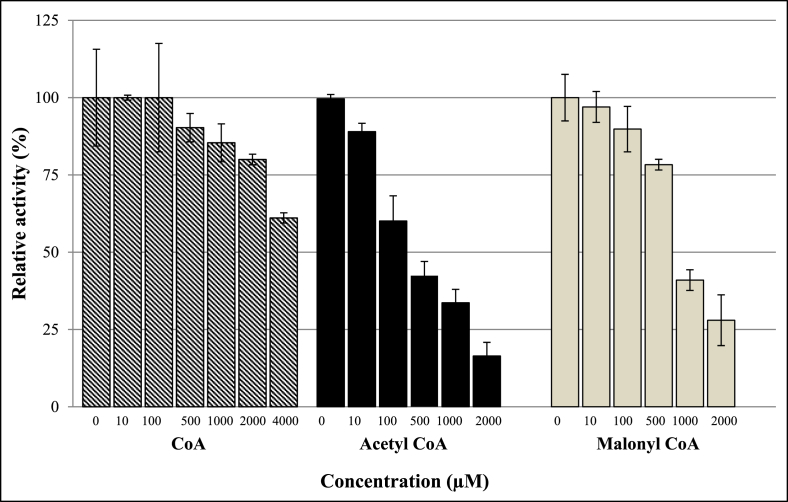
Effect of CoA, acetyl CoA, and malonyl CoA on the pantothenate kinase activities. Relative activities of EhPanK in the presence of various concentrations of inhibitors to those without inhibitors are shown. The assay was performed in 10 mM MgCl_2_, 15 mM HEPES, 20 mM NaCl, 1 mM EGTA, 0.02% Tween-20, 0.1 mg/mL γ−globulins, 0.2 mM pantothenate, and 100 μM ATP with various concentrations of CoA, acetyl CoA, or malonyl CoA at 37 °C at pH 6. The assays were carried out three times independently, and the results are shown as means ± SEM of triplicates.

**Fig. 3:**
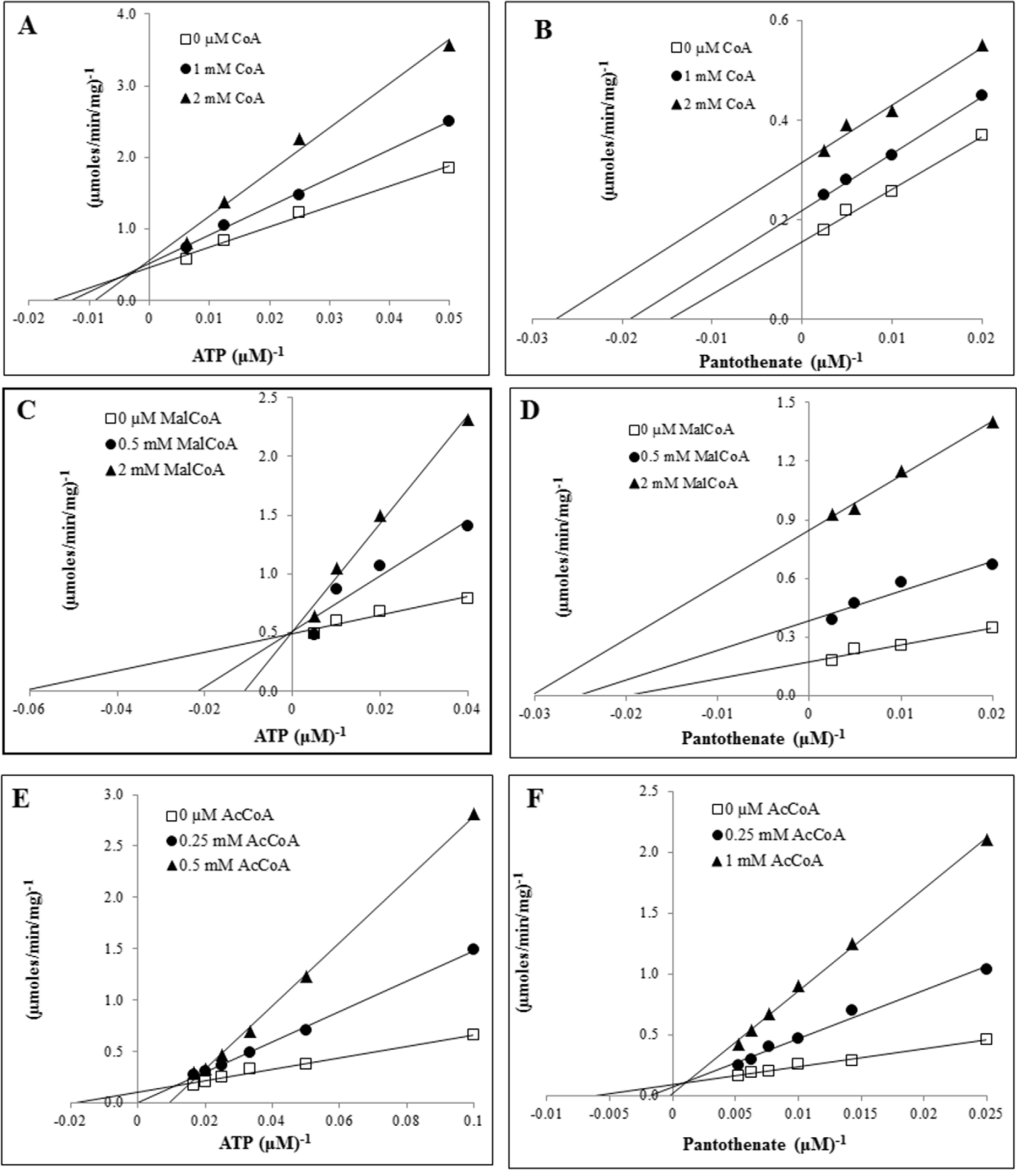
Double-reciprocal plots of the recombinant EhPanK in the presence of CoA (A and B), malonyl CoA (C and D), or acetyl CoA (E and F). The enzymatic activities were determined with various concentrations of ATP and 0.2 mM pantothenate (A, C, and E) or various concentrations of pantothenate and 100 μM ATP (B, D, and F), in the presence of three concentrations of inhibitors (0, 0.5, and 2 mM). Data are shown in means ± SEM of triplicate analyses.

PanK from other organisms was reported to be negatively regulated by allosteric inhibition with CoA, acetyl CoA, and malonyl CoA (Calder et al., 1999; Takagi et al., 2010; Vallari et al., 1987). Similarly, EhPanK was also inhibited by CoA and its derivatives, although relatively higher concentrations were needed for inhibition compared with PanK from other organisms (Brand and Strauss, 2005; Calder et al., 1999) (Fig. 2). Double-reciprocal plots revealed the mechanisms of inhibition by CoA and malonyl CoA are competitive with ATP, while inhibition by CoA and malonyl CoA are uncompetitive or non-competitive with pantothenate, respectively. The mechanisms of inhibition by acetyl CoA were not clear but seemed to be the mixed type with respect to ATP and pantothenate (Fig. 3).

### 3.7 Cellular localization of EhPanK

To examine the localization of EhPanK, we performed fractionation and immunoblot analysis of PanK using *E. histolytica* transformant-expressing Myc-tagged EhPanK. We demonstrated that EhPanK is exclusively present in the 100,000 × g supernatant fraction corresponding the cytosol (Fig. S6).

### 3.8 Effects of *EhPanK* gene silencing on growth, cellular CoA levels, and gene expression of the CoA biosynthetic pathway

**Fig. 4:**
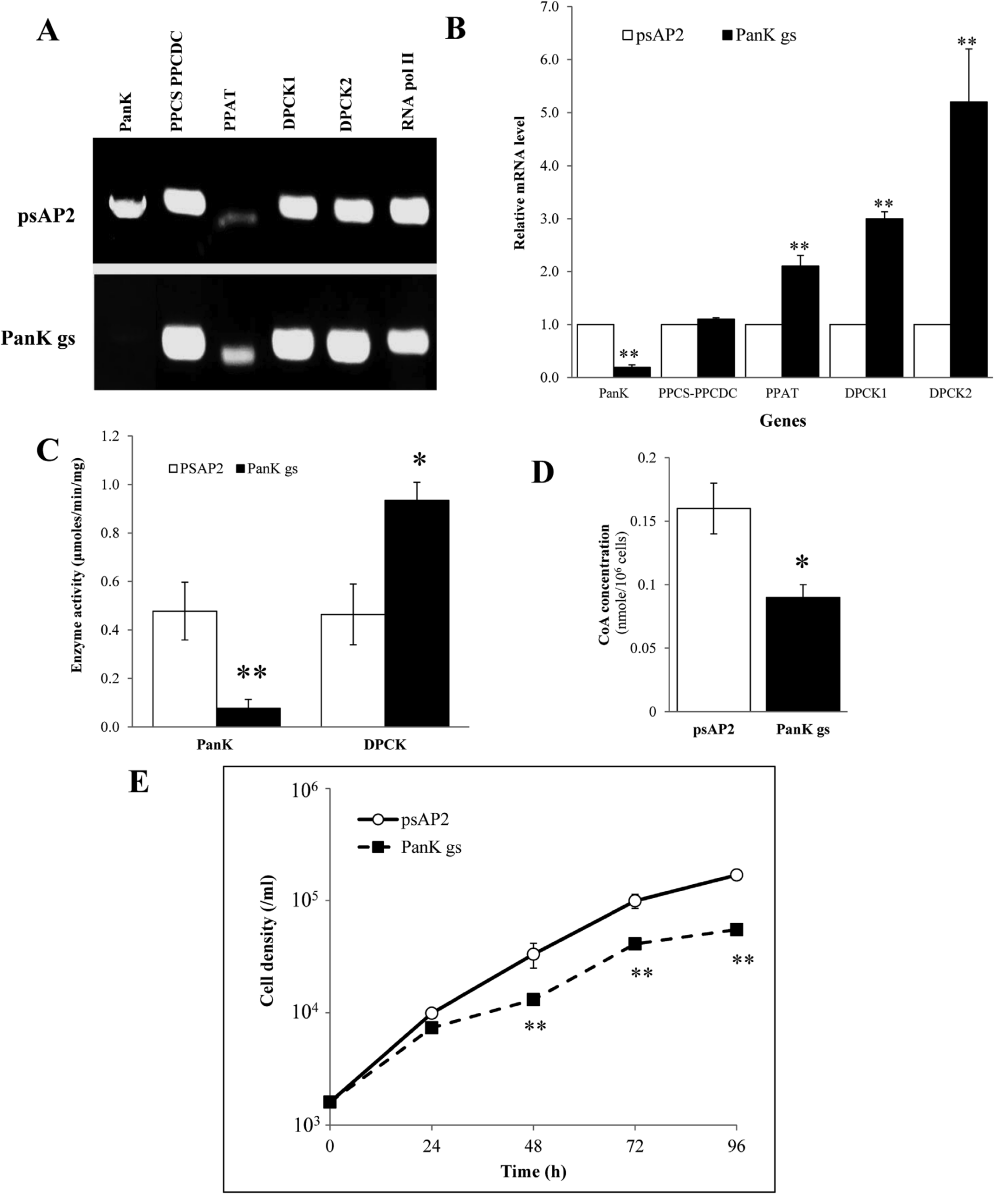
Analyses of *EhPanK* gene silenced strain. A) RT-PCR analysis of *EhPanK* gene transcript from *E. histolytica* transformants. “psAP” indicates control strain transfected with psAP-2-Gunma empty vector, and “PanK gs” indicates *EhPanK* gene silenced strain. B) Relative levels of gene transcripts encoding all four enzymes involved in CoA biosynthesis by qRT-PCR analysis in psAP and Pan Kgs transformants. qRT-PCR data were normalized against RNA polymerase II, and are shown in percentage relative to the transcript level of each gene in psAP control. C) PanK and DPCK activity in cell lysates of psAP and PanKgs transformants. D) CoA concentration in cell lysates. For B-D, data are shown in mean ± SEM of three biological replicates. Statistical comparison is made by Student's *t*-test (*P < 0.05, **P < 0.01). E) Growth kinetic of *E. histolytica* transformants during 96 h incubation in BI-S-33 medium. Data are shown in mean ± SEM of three replicates. Data from one representative experiment of three conducted independently are shown. Statistical comparison is made by Student's *t*-test (*P < 0.05, **P < 0.01).

To investigate the physiological importance and essentiality of PanK in *E. histolytica*, we created and examined an *E. histolytica* strain where *EhPanK* was silenced by antisense small RNA-mediated transcriptional gene silencing (Mirelman et al., 2008); *EhPanK* gene expression was successfully silenced (Fig. 4A). The level of silencing was estimated to be approximately 85% by qRT-PCR measurement. Interestingly, the steady-state transcript of the genes encoding other enzymes involved in CoA biosynthesis were up-regulated 1.2 ± 0.03, 2.1 ± 0.2, 3 ± 0.1, and 5.2 ± 1.0-fold for *PPCS-PPCDC, PPAT, DPCK1*, and *DPCK2*, respectively (Fig. 4B).

In order to determine whether the protein level also increased, we measured PanK and DPCK activity in cell lysates. PanK activity decreased by approximately 81% in the *EhPanK*-silenced strain compared to the control. In contrast, DPCK activity increased 2.1 fold (Fig. 4C). CoA concentration decreased by approximately 40% in *EhPanK*-silenced strain compared to the control (Fig. 4D). Growth kinetic analysis also showed that in *EhPanK*-silenced strain showed remarkable growth retardation as the cell density of *EhPanK*-silenced strain decreased to approximately 30–52% of the control at 48–96 h, respectively (Fig. 4E, Fig. S7A, Fig. S7B).

### 3.9 Identification of EhPanK inhibitors from Kitasato Natural Products Library

**Fig. 5:**
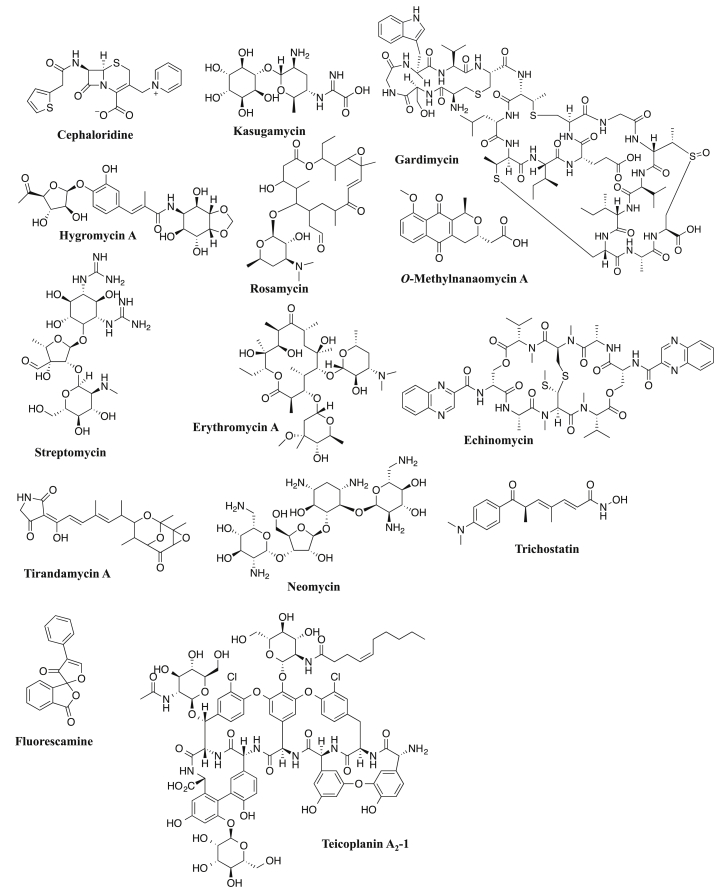
Structures of *EhPanK* inhibitors identified in this study.

Based on the significant biological role of EhPanK, inhibitors of this enzyme should be considered to possess properties needed for novel anti-amoebic agents. Therefore, we conducted enzyme-based screening of the Kitasato Natural Products Library composed of 244 structurally elucidated compounds using recombinant EhPanK to identify potential PanK inhibitors. We identified 14 compounds (Fig. 5) that demonstrated an IC_50_ value of <400 μM (Table 4, Fig. S8). Among these compounds, Teicoplanin had the best inhibitory activity against EhPanK with an IC_50_ value of 26.3 ± 2.6 μM. All EhPanK inhibitors also inhibited *E. histolytica* trophozoites although the IC_50_ values of most of the identified EhPanK inhibitors were high. Six compounds, *O*-methylnanaomycin A, Echinomycin, Tirandamycin A, Neomycin, Trichostatin, and Teicoplanin, showed the IC_50_ values of <20 μM against *E. histolytica* trophozoites, but the IC_50_ values of *O*-methylnanaomycin A, Echinomycin, Tirandamycin A, Neomycin, and Trichostatin against *E. histolytica* trophozoites were lower than their IC_50_ values against EhPanK activity, suggesting that the target of these compounds for growth inhibition are unlikely to be through PanK inhibition. In contrast, Teicoplanin demonstrated comparable IC_50_ values against PanK activity and *E. histolytica* growth (Table 5).

**Table 4:** Inhibitory activities of EhPanK inhibitors from Kitasato Natural Products Library.

Compound	IC_50_ (μM)
Cephaloridine	229.9 ± 10.1
Kasugamycin	254.1 ± 4.7
Gardimycin	49.2 ± 4.8
Hygromycin A	193.7 ± 17.8
*O*-methylnanaomycin A	283.8 ± 28.7
Rosamycin	165.7 ± 2.0
Streptomycin	155.4 ± 10.1
Erythromycin A	131.3 ± 6.0
Echinomycin	77.8 ± 4.4
Tirandamycin A	228.8 ± 3.4
Neomycin	147.2 ± 6.8
Fluorescamine	346.4 ± 14.7
Trichostatin	309.9 ± 14.9
Teicoplanin	26.3 ± 2.6

Mean ± SEM of three replicates are shown.

**Table 5:** *In vitro* anti-amebic activity and cytotoxicity against MRC-5 cells of EhPanK inhibitors.

Compound	IC_50_ (μM)	
	*E. histolytica* trophozoite	Normal human cell (MRC-5)
Cephaloridine	245.78 ± 34.92	48.20 ± 3.61
Kasugamycin	66.16 ± 13.68	87.10 ± 7.22
Gardimycin	34.40 ± 3.16	>52.9
Hygromycin A	142.22 ± 16.34	>196
*O*-methylnanaomycin A	1.68 ± 0.05	18.98 ± 0.49
Rosamycin	56.34 ± 11.72	34.40 ± 2.71
Streptomycin	77.53 ± 1.52	>172
Erythromycin A	155.88 ± 20.57	>136
Echinomycin	0.26 ± 0.04	<0.38
Tirandamycin A	13.71 ± 4.85	143.88 ± 3.80
Neomycin	10.58 ± 1.12	>163
Fluorescamine	167.90 ± 20.07	287.77 ± 17.84
Trichostatin	0.29 ± 0.04	2.62 ± 0.49
Teicoplanin	15.08 ± 2.25	>53.3
Metronidazole	0.66 ± 0.05	>100

Mean ± SEM of three replicates are shown.

## 4 Discussion

### 4.1 Identification of CoA biosynthesis as rational drug target

CoA is an essential and ubiquitous cofactor functioning as an acyl carrier as well as a carbonyl activating group for many metabolic reactions. In the present study, we identified four enzymes that are involved in CoA synthesis in *E. histolytica* (Fig. 1) and characterized the first rate-limiting enzyme in this process, PanK. We have provided evidence for the pivotal role of this enzyme in cell proliferation by gene silencing.

Since all genes involved in CoA biosynthesis are transcribed in all amoeba life cycle stages, this pathway is the good target not only for killing the pathogenic trophozoites but inhibiting stage conversion and thus amoeba transmission. The mechanism coordinating gene expression regulation among the five genes involved in *E. histolytica* CoA biosynthesis remains elusive. However, based on our previous transcriptomic analysis of *E. invadens*, a related reptilian amoeba species that also causes an invasive disease and has been used as a model system for *E. histolytica* (Donaldson et al., 1975; Kojimoto et al., 2001; Chia et al., 2009) during encystation (De Cádiz et al., 2013), we found that PanK is transcribed in both trophozoite and encystation life cycle stages at comparable levels, suggesting that PanK is required in both stages.

A *EhPanK* gene-silenced strain was successfully established, after producing a strain in which *EhPanK* gene expression was ∼85% repressed. Four failed attempts to create of such a cell line (data not shown) suggest the essentiality of this gene. The low levels of EhPanK in our experiment can keep the cells viable, consistent with the previous publication in *Mycobacterium tuberculosis* PanK (Reddy et al., 2014). Interestingly, *EhPanK* gene silencing resulted in the simultaneous transcriptional up-regulation of genes for downstream CoA pathway enzymes. Remarkably, such genes, DPCK1 and DPCK2, were up-regulated 3- and 5-fold, respectively. This observation is similar to that in experimentation with *Plasmodium yoelii*, where other genes in the CoA biosynthetic pathway were up-regulated when *PanK2* gene was knocked out (Hart et al., 2016). Despite the apparent compensatory upregulation of downstream enzyme genes, CoA concentration was significantly reduced, which is most likely a direct cause of decreased ATP generation and retarded growth.

In order to validate EhPanK as the drug target, there should be notable biochemical, structural, and genetic differences in their human homologs. Such characteristics should, in theory, minimize toxic side effects of pharmacological inhibition of human enzymes via amebiasis chemotherapy. *E. histolytica* has one PanK enzyme whereas *Homo sapiens* has five PanK isoforms encoded by four genes. EhPanK is most closely related to human PanK4 with 35% amino acid identity, but phylogenetic analyses suggest a distant relationship between EhPanK and its human counterparts.

While PanK is indispensable for the optimal growth of *E. histolytica* trophozoites, but in some eukaryotes, including human, PanK was shown non-essential. It has been demonstrated that human cells have the ability to hydrolyze exogenous CoA to 4′-phosphopantetheine by ectonucleotide pyrophosphatase, and then incorporate 4′-phosphopantetheine and enzymatically convert it back to CoA by the bifunctional enzyme CoA synthase (Srinivasan et al., 2015). In *Plasmodium*, it was reported that PanK is nonessential in blood stage parasite development, but essential in the hepatic stage, as well as oocyst and sporozoite formation in the mosquito (Hart et al., 2016; Srivastava et al., 2016). In *M. tuberculosis*, CoaA was proven to be an essential enzyme (Awasthy et al., 2010), but was also shown in *in vitro* and *in vivo* studies to be a suboptimal target for antimycobacterial drug development because expression of *CoaA* gene needs to be repressed to obtain sufficient cidal effects by inhibitors (Reddy et al., 2014). On the other hand, CoaBC, a bifunctional enzyme in the CoA biosynthesis, has proven to be essential, but its suitability as drug target needs to be chemically validated in future (Evans et al., 2016). Inhibition of PanK in human cells may be tolerated, which also supports the premise that PanK is a reasonable chemotherapeutic target against pathogens for humans.

### 4.2 Hit discovery of EhPanK inhibitors

Historically, natural products, especially secondary metabolites, have provided various drugs against human diseases (Newman and Cragg, 2012). Our group has recently discovered several natural compounds from fungi and actinomycetes that inhibit cysteine synthase, an enzyme absent in humans and involved in *de novo* biosynthesis of L-cysteine (Mori et al., 2015). In this study, we identified 14 inhibitors of EhPanK; half of the inhibitors have sugar moieties, and some of them are aminoglycosides.

Teicoplanin showed moderate inhibitory activity with IC_50_ 26.3 ± 2.6 μM and inhibition of *E. histolytica* trophozoite growth (IC_50_ 15.1 ± 2.3 μM). Although Teicoplanin also demonstrated cytotoxicity to MRC-5 cells, preference towards *E. histolytica* cells was identified. Teicoplanin is a mixture of glycopeptide antibiotics used against Gram-positive bacteria including methicillin-resistant *Staphylococcus aureu*s, with a mechanism of action inhibiting bacterial cell wall synthesis. Teicoplanin consists of the glycopeptide core, two carbohydrates (mannose and *N*-acetylglucosamine), and a side chain. The components of teicoplanin are classified by their side chain length and conformation; the major components are A_2_-1 through A_2_-5.

In conclusion, we have shown that pantothenate kinase and CoA biosynthesis are physiologically important for this parasite. We have also shown that potential EhPanK inhibitors were identified by screening the natural product library, suggesting that the enzyme can be a new target for the development of anti-amebic drugs.

## Appendix A Supplementary data

**Fig. S1**. Relative mRNA expression level of all enzymes in CoA biosynthesis. A) Relative levels in trophozoites of the *E. histolytica* HM1: IMSS cl6 (Penuliar et al., 2012, 2015) and G3 (Furukawa et al., 2013, 2012; Nakada-Tsukui et al., 2012) strain from array data. B) Relative levels in *E. invadens* encystation stage. Data was generated from the previous publication (De Cádiz et al., 2013).

**Fig. S2**. Phylogenetic tree of PanK from *E. histolytica* and other species. Optimal ML tree inferred by RAxML program with LG +Γ4 model was shown. Unambiguously aligned 145 positions from 81 sequences were used for the analysis.Branch lengths are proportional to estimated numbers of substitutions. Bootstrap proportion (BP) values (more than 50) of the rapid bootstrap analysis are shown on the internal branches. For each of the bacterial species, names a taxonomic group ID for Firmicutes (B31) in Table S1 is also indicated.

**Fig. S3**. Alignment of PanK protein sequences from *E. histolytica* and other organisms. The sequences were aligned using Muscle program (Edgar, 2004) in SeaView package version 4.6.1 (Gouy et al., 2010). The species names and the NCBI accession numbers are as follows: *E. histolytica* (XP_001913460); *Entamoeba nuttali* (XP_008855986); *Homo sapiens* PanK4 (NP_060686); *Homo sapiens* PanK3 (NP_078870); *Arabidopsis thaliana* (NP_001031768). Identical residues are denoted by asterisks (*). The glycine residue in the ATP binding sites are shown in the white letter on with a black background. Serine, arginine, and alanine residues highlighted with black letters on a grey background indicate residues implicated in pantothenate binding sites.

**Fig. S4**. Expression and purification of EhPanK in *E. coli*. A) Expression and purification of recombinant EhPanK. Protein samples at each step of purification were subjected to 12% SDS-PAGE under reducing conditions, and the gel was stained with Coomassie Brilliant Blue. B) Immunoblot analysis of purified recombinant EhPanK using anti His-Tag antibody.

**Fig. S5**. Optimum pH and temperature (A) and initial rate plots (B, C) of EhPanK. A) Enzyme specific activity of recombinant EhPanK was measured at the various pHs and temperatures indicated in the figure. B) The velocity of EhPanK reaction at various pantothenate concentrations in the presence of 25, 50, or 100 μM ATP. C). The velocity of EhPanK reaction at various ATP concentrations in the presence of 8, 64, or 128 μM pantothenate. Assays were performed as described in Materials and methods, with 50 ng of EhPanK recombinant enzyme, 15 mM HEPES, 20 mM NaCl, 10 mM MgCl_2_, 1 mM EGTA, 0.02% Tween-20, 0.1 mg/mL β−globulins. Assays for panes B and C were conducted at 37 °C at pH 6. Data are shown in mean ± SEM of three replicates.

**Fig. S6**. Cellular fractionation and immunoblot analysis of EhPanK. Trophozoites of Myc-EhPanK expressing transformant (Myc-PanK) were fractionated as described in Materials in Methods and subjected to immunoblot analysis using anti-Myc monoclonal antibody, anti-CPBF1, and anti-CS1 polyclonal antisera. CPBF1 and CS1 serve as a control of organelle and cytosolic proteins, respectively. The whole lysate of control strain transformed with empty vector was also subjected to analysis.

**Fig. S7**. Growth kinetics of *E. histolytica* transformants during 96 h incubation in BI-S-33 medium from two other independent experiments (A and B). Data are shown in mean ± SEM of three replicates.

**Fig. S8**. Dose-response data of inhibitors against EhPanK inhibitors. IC50 values were determined by dose-response-inhibition using GraphPad Prism (GraphPad Software Inc., San Diego, USA). Data are shown in mean of three replicates.

mmc1 mmc1

mmc2 mmc2

Table S1 Detail information of pantothenate kinase from various organisms related to *E. histolytica* that used in phylogenetic analysis. Table S1

Table S2 Purification of recombinant *E. histolytica* pantothenate kinase. Enzyme activity was measured as described in Materials and methods. Table S2
