# Supplementary material for: Characterization and validation of Entamoeba histolytica pantothenate kinase as a novel anti-amebic drug target
Source: Int J Parasitol Drugs Drug Resist. 2018 Mar 1;8(1):125–36. doi: 10.1016/j.ijpddr.2018.02.004 (PMC6114107; doi:10.1016/j.ijpddr.2018.02.004)
Supplement: mmc1 [file mmc1.pdf]

Fig. S1

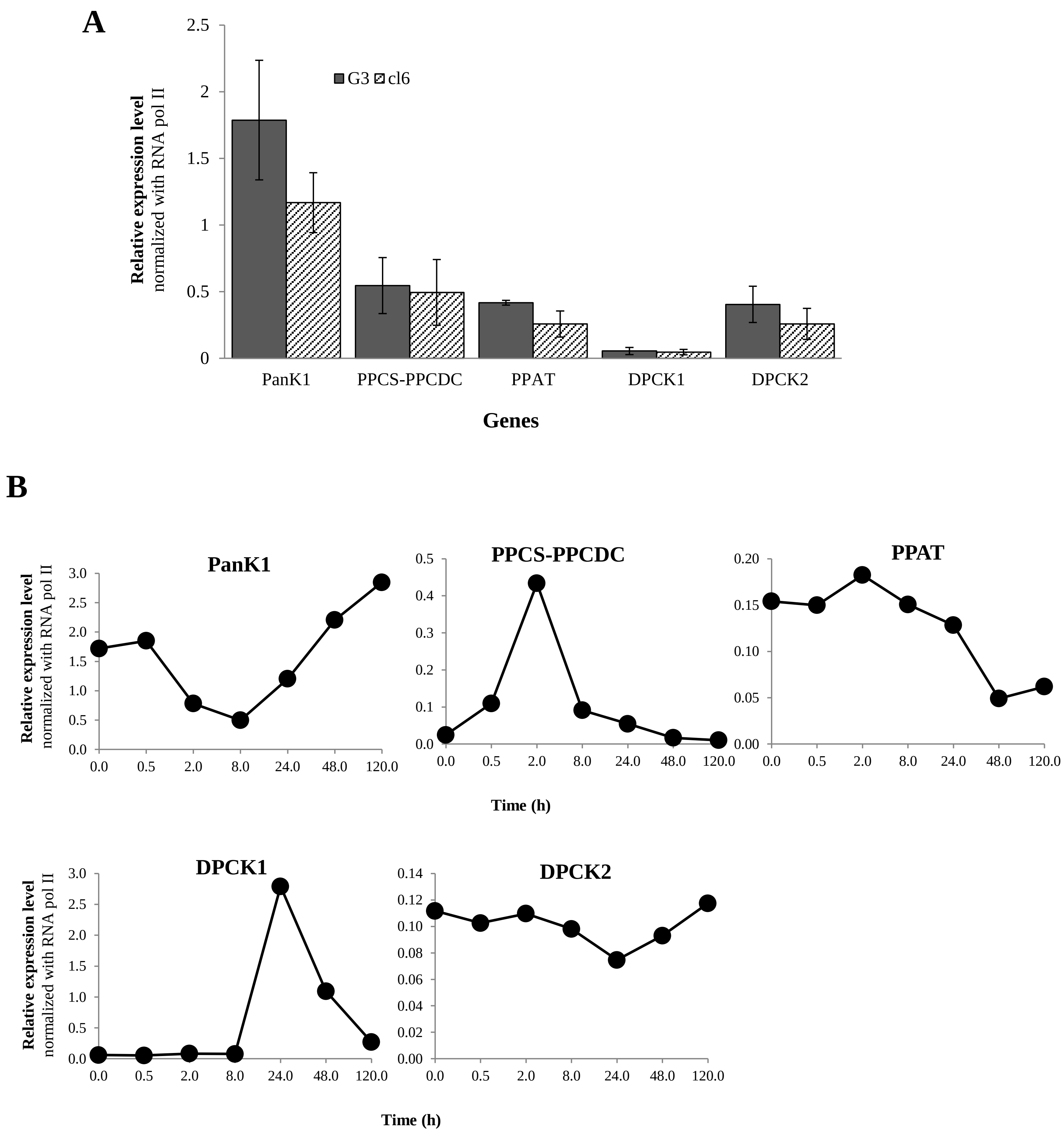

A
Relative expression level
normalized with RNA pol II
G3
cl6
PanK1
PPCS-PPCDC
PPAT
DPCK1
DPCK2
Genes
B
PanK1
PPCS-PPCDC
PPAT
DPCK1
DPCK2
Time (h)

Fig. S3

```
E.histolytica         -------------------------------MSQPSHSIPSKHSVLSNEDIE-HDDHITH    28
E.nuttali             -------------------------------MSQPSHSIPSKHDVLFNEDIE-HDDHITH    28
H.sapiens PanK4       -----------------------MSWPGTFKMAEC--G----AS-GSGS---------SG    21
H.sapiens PanK3       ------------------------------------------------------------     0
A.thaliana PanK2      MAGQEDEYDPILDNKREAEAKSQVSVAADKNMAPSTSGTPIHRS-GSRPQLDLSKAEIQG    59

E.histolytica         TFEQYEFTKTQSPLPLNGVRRRTAPLSADMGGTMTKIVFWIPGDTDIDLPK-YVEPEESV    87
E.nuttali             NFEQYEFTKTQSPLPLNGVRRRTAPLSADIGGTMTKIVFWIPGDTDIDLPK-YVEPEESV    87
H.sapiens PanK4       --DSLDKSITLPPDEIFRNLENAKRFAIDIGGSLTKLAYYSTVQHKVAKVRSFDHSGKDT    79
H.sapiens PanK3       ------------MKIKDAKKPSFPWFGMDIGGTLVKLSYFEPIDITAEEEQEEVESLKSI    48
A.thaliana PanK2      NLEERDPTILL-----PNQSDDISHLALDIGGSLIKLLYFSRHEDYSNDDDKRKRT--IK   112
                                                  * **   *

E.histolytica         --------MSGFDLYPD--PVLKVTMNSKGENELCFMKFPTSQIPEFIHYAKTSKLKEI-   136
E.nuttali             --------MSGFDLYPD--PVLKVTMNSKGENELCFMKFPTLQIPEFIHYAKTSKLKEI-   136
H.sapiens PanK4       ----------EREHEPP--YEISVQ--EEITARLHFIKFENTYIEACLDFIKDHLVN---   122
H.sapiens PanK3       RKYLTSNVAYGSTGIRDVHLELKDLTLFGRRGNLHFIRFPTQDLPTFIQMGRDKNF----   104
A.thaliana PanK2      ----------ERLGITN--GNLRSY—PVLGGRLHFVKFETHKINECLDFIHSKQLHRRDP   159

E.histolytica         -YG------IGNLQILNVTGGGAYKFANLLKDEMDLEVRQQDEMRCLVLGVNFLLLCC--   187
E.nuttali             -YG------IGNLQILNVTGGGAYKFANLLKDEMDLEVRQQDEMRCLVLGVNFLLLCC--   187
H.sapiens PanK4       ----------TETKVIQATGGGAYKFKDLIEEKLRLKVDKEDVMTCLIKGCNFVLKNI--   170
H.sapiens PanK3       ---------STLQTVLCATGGGAYKFEKDFRTIGNLHLHKLDELDCLVKGLLYIDSVSFN   155
A.thaliana PanK2      PYPWSSKTLPLGTGVIKVTGGGAFKFADLFKERLGVSIEKEDEMHCLVSGANFLLKAI--   217
                                        ***** **               *   *** *

E.histolytica         QEKLCFRFVNKTKL-----YREVTPEIFPYLLVNVGSGVSILKVTAPEKYERVSGSLIGG   242
E.nuttali             QEKLCFRFVNKTKL-----YREVTPEIFPYLLVNVGSGVSILKVTAPEKYERVSGSLIGG   242
H.sapiens PanK4       -PHEAFVYQKDSD--PEFRFQTNHPHIFPYLLVNIGSGVSIVKVETEDRFEWVGGSSIGG   227
H.sapiens PanK3       GQAECYYFANASEPERCQKMPFNLDDPYPYLLVNIGSGVSILKVHSKDNYKRVTGTSLGG   215
A.thaliana PanK2      -RHEAFTHMEGEK--EF--VQIDPNDLYPYLLVNVGSGVSIIKVDGEGKFERVSGTNVGG   272
                                                  ****** ****** *       * * *   **

E.histolytica         GTFWGLCCLLTNYKSFDEMLQAAQYGQHQNVDLYVKDIYGR-CYDSVGLAADMMASSFGK   301
E.nuttali             GTFWGLCCLLTNYKSFDEMLQAAQYGQHQNVDLYVKDIYGR-CYDSVGLAADMMASSFGK   301
H.sapiens PanK4       GTFWGLGALLTKTKKFDELLHLASRGQHSNVDMLVRDVYGGAH-QTLGLSGNLIASSFGK   286
H.sapiens PanK3       GTFLGLCSLLTGCESFEEALEMASKGDSTQADKLVRDIYGG-DYERFGLPGWAVASSFGN   274
A.thaliana PanK2      GTYWGLGRLLTKCKSFDELLELSQKGDNSAIDMLVGDIYGGMDYSKIGLSASTIASSFGK   332
                      **  **  ***    * * *           *    * **       **     *****

E.histolytica         IQKVPCGEAKNHYSEADIAQGLMMMVCGSIAHLGFELTKLHHINRIFYTGGFVAHNPVVW   361
E.nuttali             VQKVPCGEAKNHYSEADIAQGLMMMVCGSIAHLGFELTKLHHINRIFYTGGFVAHNPVVW   361
H.sapiens PanK4       SATA--DQ---EFSKEDMAKSLLHMISNDIGQLACLHARLHSLDRVYFGGFFIRGHPVTM   341
H.sapiens PanK3       MIYK--EK-RESVSKEDLARATLVTITNNIGSVARMCAVNEKINRVVFVGNFLRVNTLSM   331
A.thaliana PanK2      AISE--NKELDDYRPEDISLSLLRMISYNIGQISYLNALRFGLKRIFFGGFFIRGHAYTM   390
                                      *            *              *    * *

E.histolytica         EAITTMLTTWSSGSVDANFLEYDGYFGSIGALLLSEYEEE--LM----------------   403
E.nuttali             EAITTMLTTWSSGSVDANFLEYDGYFGSIGALLLSEYEEE--LM----------------   403
H.sapiens PanK4       RTITYSINFFSKGEVQALFLRHEGYLGAIGAFLKGAEQDNP--------NQYSWGENYAG   393
H.sapiens PanK3       KLLAYALDYWSKGQLKALFLEHEGYFGAVGALLGLPNFS---------------------   370
A.thaliana PanK2      DTISFAVHFWSKGEMQAMFLRHEGFLGALGAFMSYEKHGLDDLMSHQLVERFPMGAPYTG   450
                                * *   * **   *  *  **
```

Fig. S4

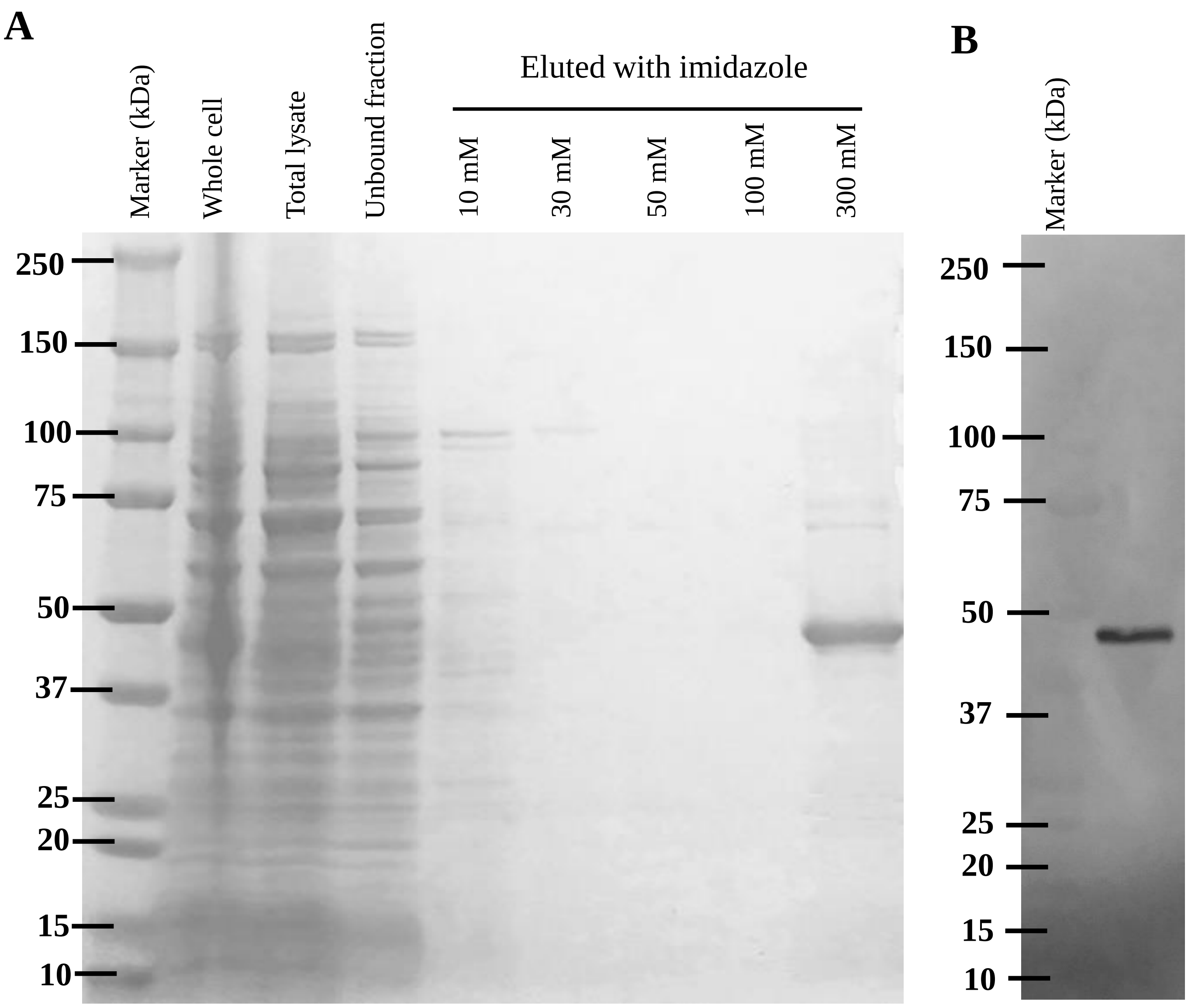
A
B
Eluted with imidazole
Marker (kDa)
Whole cell
Total lysate
Unbound fraction
10 mM
30 mM
50 mM
100 mM
300 mM
250
150
100
75
50
37
25
20
15
10
Marker (kDa)
250
150
100
75
50
37
25
20
15
10

Fig. S5

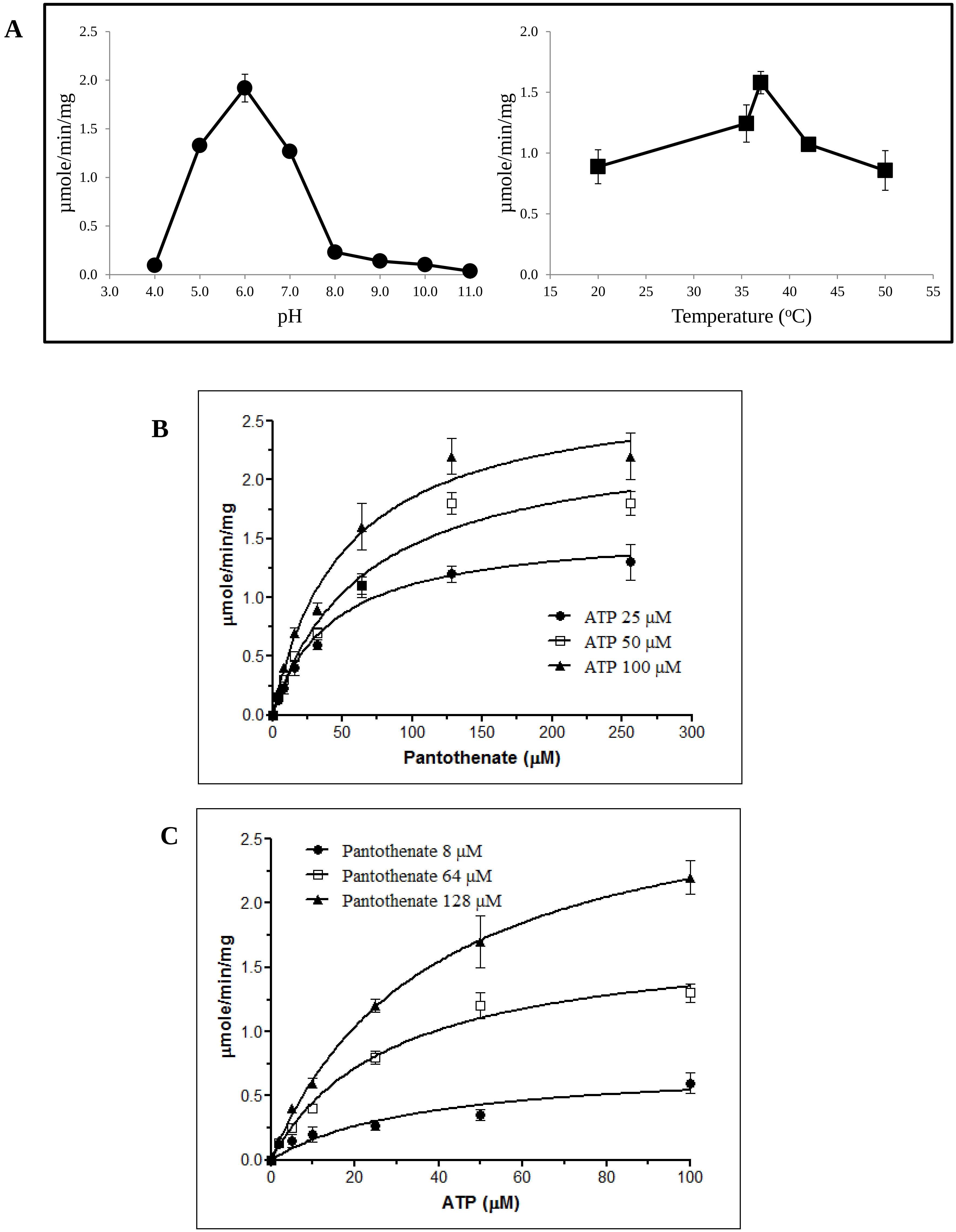
A
µmole/min/mg
pH
µmole/min/mg
Temperature (°C)
B
µmole/min/mg
Pantothenate (µM)
ATP 25 µM
ATP 50 µM
ATP 100 µM
C
µmole/min/mg
ATP (µM)
Pantothenate 8 µM
Pantothenate 64 µM
Pantothenate 128 µM

Fig. S6

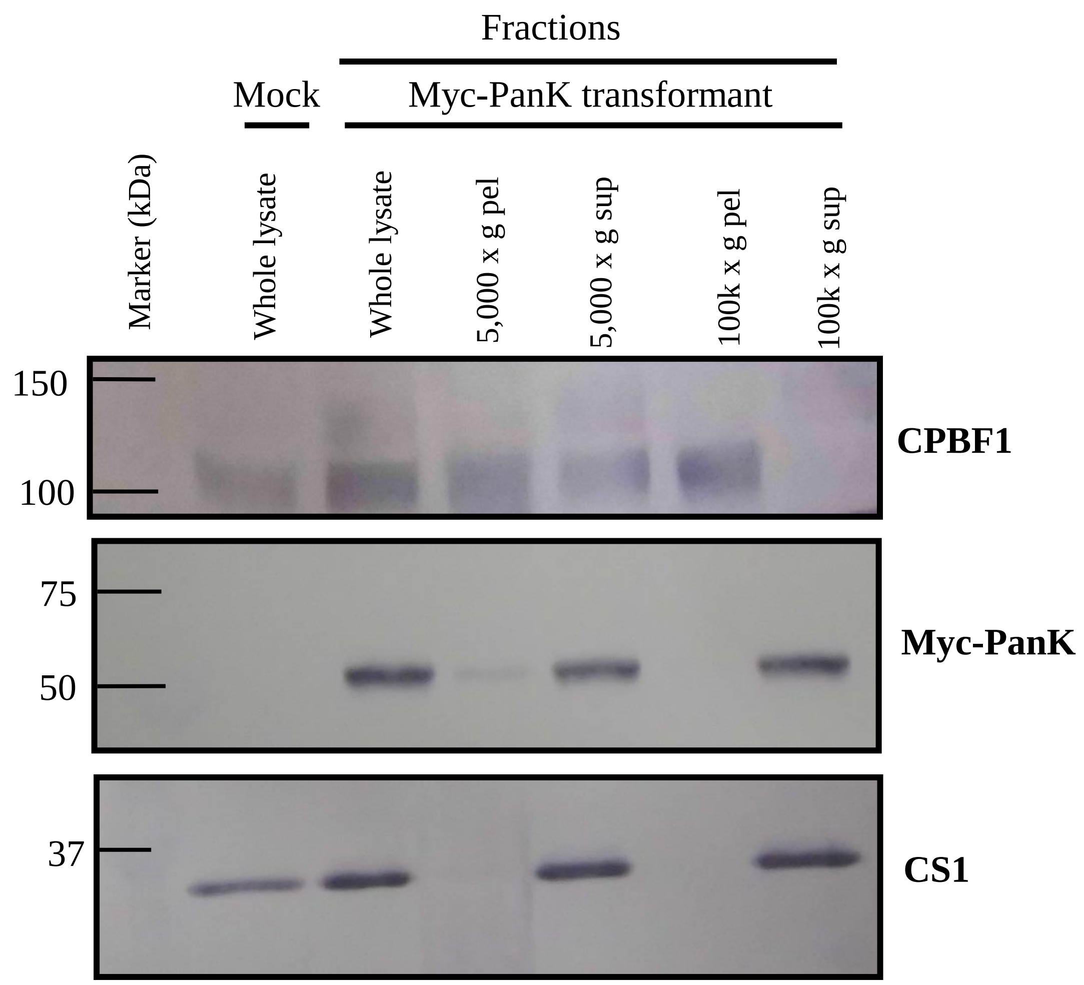

Fractions
Mock
Myc-PanK transformant
Marker (kDa)
Whole lysate
Whole lysate
5,000 x g pel
5,000 x g sup
100k x g pel
100k x g sup
150
100
CPBF1
75
50
Myc-PanK
37
CS1

Fig. S7

A

B

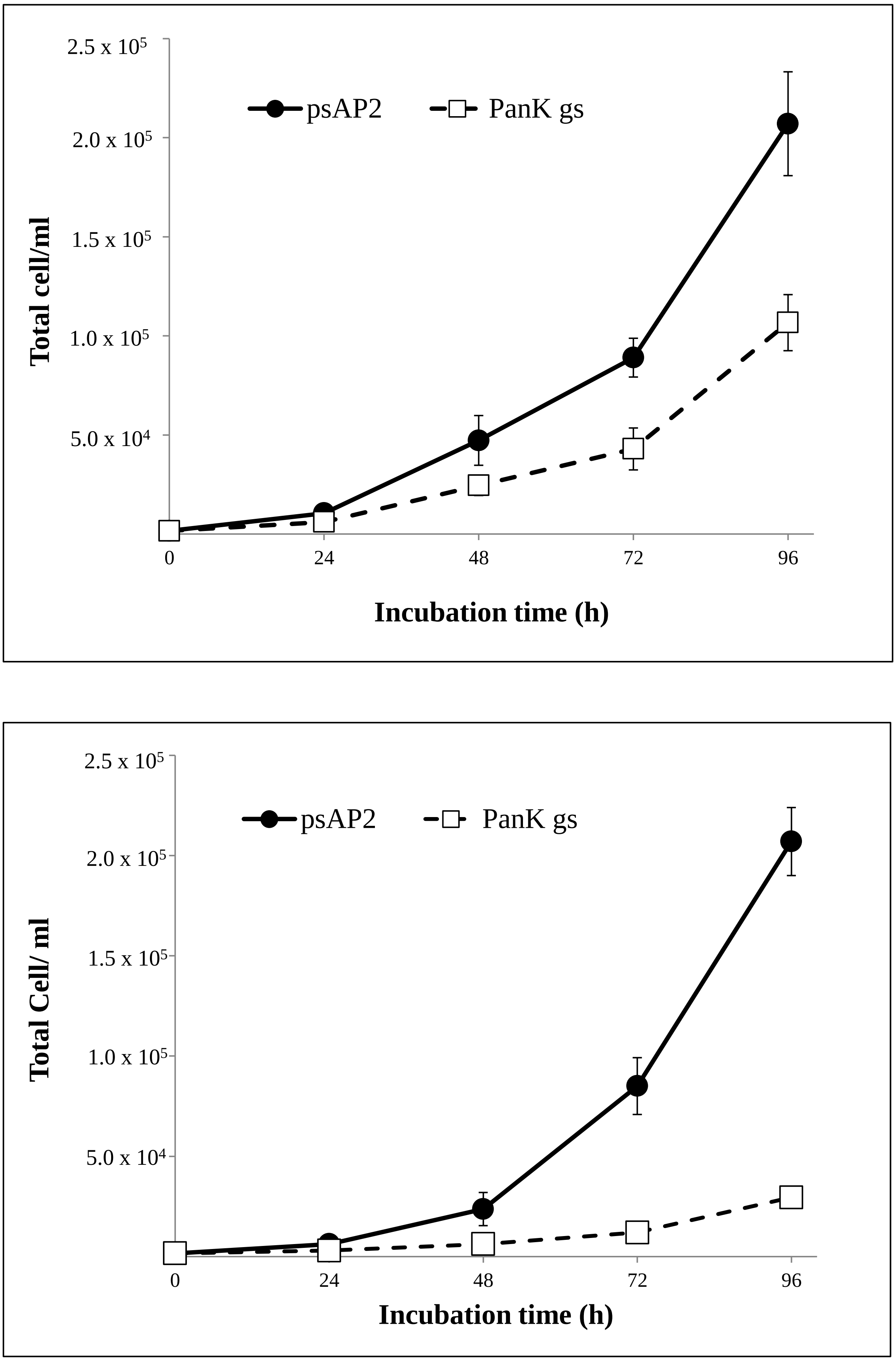
2.5 x 10^5
2.0 x 10^5
1.5 x 10^5
1.0 x 10^5
5.0 x 10^4
psAP2
PanK gs
Total cell/ml
0
24
48
72
96
Incubation time (h)
2.5 x 10^5
2.0 x 10^5
1.5 x 10^5
1.0 x 10^5
5.0 x 10^4
psAP2
PanK gs
Total Cell/ ml
0
24
48
72
96
Incubation time (h)

Fig. S8

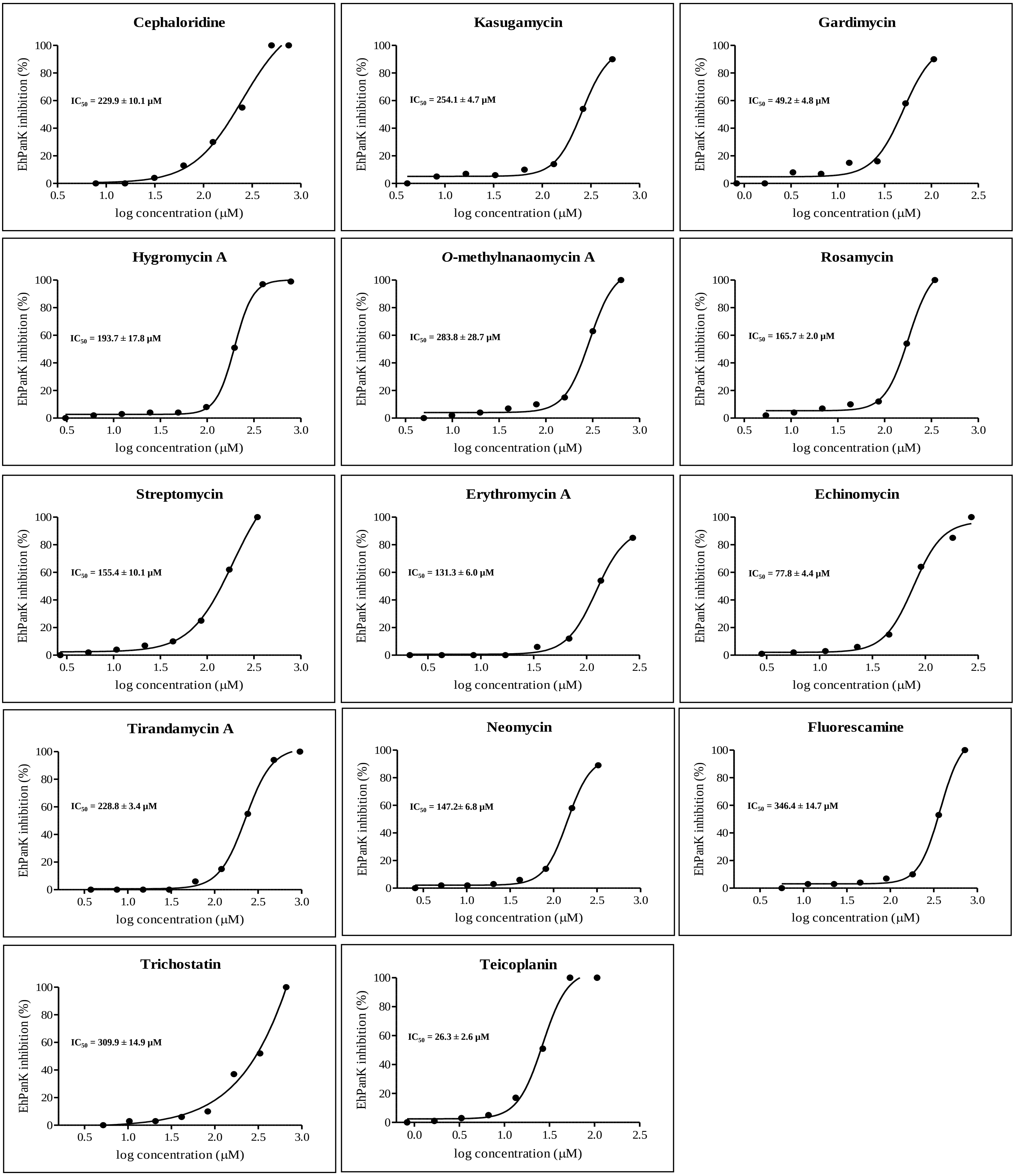
Cephaloridine
IC50 = 229.9 ± 10.1 μM
Kasugamycin
IC50 = 254.1 ± 4.7 μM
Gardimycin
IC50 = 49.2 ± 4.8 μM
Hygromycin A
IC50 = 193.7 ± 17.8 μM
O-methylnanaomycin A
IC50 = 283.8 ± 28.7 μM
Rosamycin
IC50 = 165.7 ± 2.0 μM
Streptomycin
IC50 = 155.4 ± 10.1 μM
Erythromycin A
IC50 = 131.3 ± 6.0 μM
Echinomycin
IC50 = 77.8 ± 4.4 μM
Tirandamycin A
IC50 = 228.8 ± 3.4 μM
Neomycin
IC50 = 147.2± 6.8 μM
Fluorescamine
IC50 = 346.4 ± 14.7 μM
Trichostatin
IC50 = 309.9 ± 14.9 μM
Teicoplanin
IC50 = 26.3 ± 2.6 μM
EhPanK inhibition (%)
log concentration (μM)
