# Supplementary figures and images for: Characterization and validation of Entamoeba histolytica pantothenate kinase as a novel anti-amebic drug target
Source: Int J Parasitol Drugs Drug Resist. 2018 Mar 1;8(1):125–36. doi: 10.1016/j.ijpddr.2018.02.004 (PMC6114107; doi:10.1016/j.ijpddr.2018.02.004)

Fig. S2

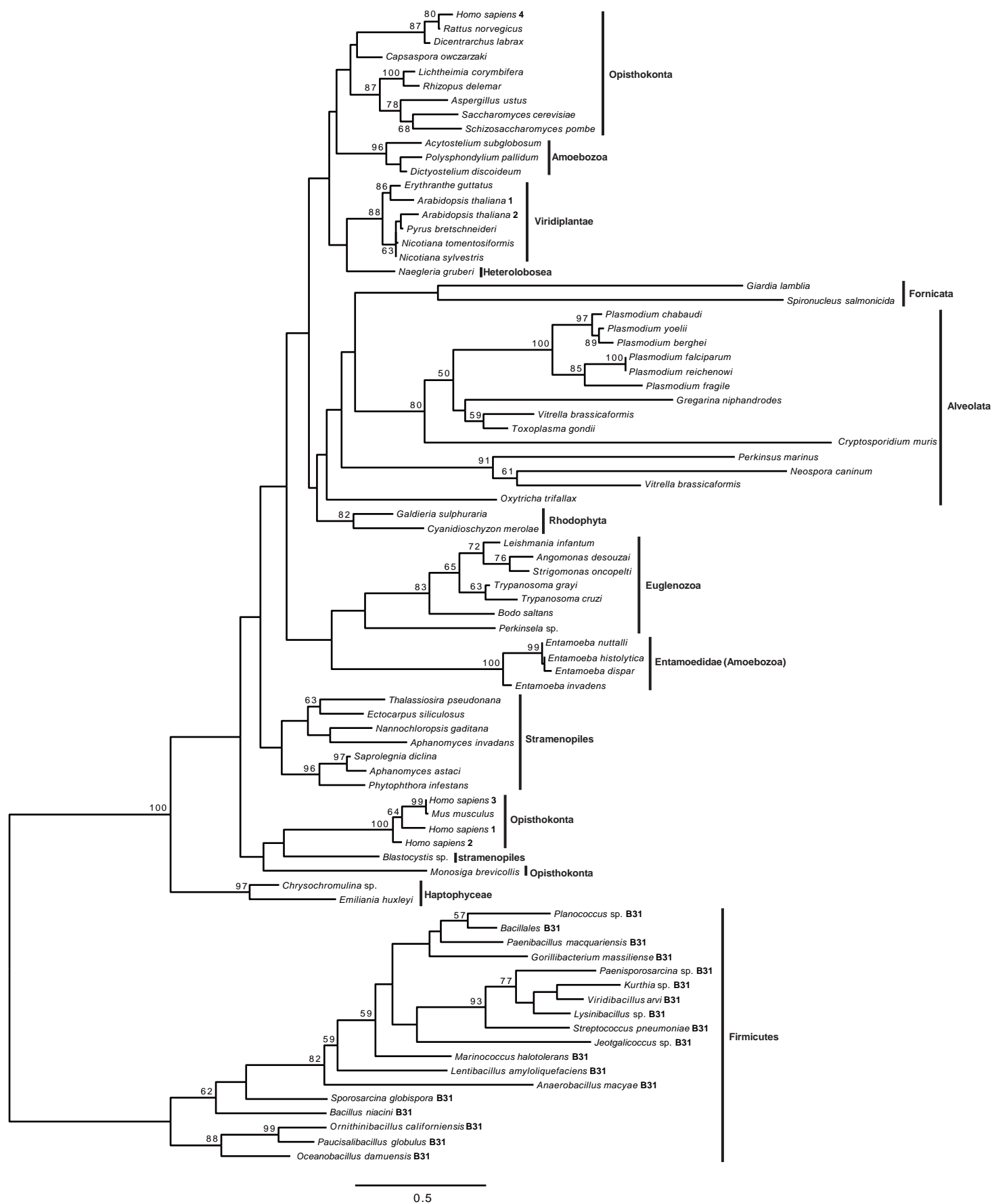

Supplement: mmc2 [file mmc2.pdf]
