# Supplementary material for: Characterization and validation of Entamoeba histolytica pantothenate kinase as a novel anti-amebic drug target
Source: Int J Parasitol Drugs Drug Resist. 2018 Mar 1;8(1):125–36. doi: 10.1016/j.ijpddr.2018.02.004 (PMC6114107; doi:10.1016/j.ijpddr.2018.02.004)
Supplement: Table S2 — Purification of recombinant E. histolytica pantothenate kinase. Enzyme activity was measured as described in Materials and methods. [file mmc3.docx]

**Supplementary Table 2.**

| **Sample** | **Protein concentration (mg/ 500 mL culture)** | **Total activity (μmole/min)** | **Specific activity (μmole/min/mg)** | **Yield (%)** | **Purification (fold)** |
| --- | --- | --- | --- | --- | --- |
| Whole lysate | 4.26 | 1.11 | 0.26 | 100 | - |
| Eluate | 0.25 | 0.39 | 1.59 | 35.2 | 6.1 |
